# Comparative analysis of KRAS4a and KRAS4b splice variants reveals distinctive structural and functional properties

**DOI:** 10.1126/sciadv.adj4137

**Published:** 2024-02-14

**Authors:** Matthew J. Whitley, Timothy H. Tran, Megan Rigby, Ming Yi, Srisathiyanarayanan Dharmaiah, Timothy J. Waybright, Nitya Ramakrishnan, Shelley Perkins, Troy Taylor, Simon Messing, Dominic Esposito, Dwight V. Nissley, Frank McCormick, Andrew G. Stephen, Thomas Turbyville, Gabriel Cornilescu, Dhirendra K. Simanshu

**Affiliations:** ^1^ NCI RAS Initiative, Cancer Research Technology Program, Frederick National Laboratory for Cancer Research, Frederick, MD, USA.; ^2^ Helen Diller Family Comprehensive Cancer Center, University of California, San Francisco, 1450 3rd Street, San Francisco, CA, USA.

## Abstract

*KRAS*, the most frequently mutated oncogene in human cancer, produces two isoforms, KRAS4a and KRAS4b, through alternative splicing. These isoforms differ in exon 4, which encodes the final 15 residues of the G-domain and hypervariable regions (HVRs), vital for trafficking and membrane localization. While KRAS4b has been extensively studied, KRAS4a has been largely overlooked. Our multidisciplinary study compared the structural and functional characteristics of KRAS4a and KRAS4b, revealing distinct structural properties and thermal stability. Position 151 influences KRAS4a’s thermal stability, while position 153 affects binding to RAF1 CRD protein. Nuclear magnetic resonance analysis identified localized structural differences near sequence variations and provided a solution-state conformational ensemble. Notably, *KRAS4a* exhibits substantial transcript abundance in bile ducts, liver, and stomach, with transcript levels approaching *KRAS4b* in the colon and rectum. Functional disparities were observed in full-length KRAS variants, highlighting the impact of HVR variations on interaction with trafficking proteins and downstream effectors like RAF and PI3K within cells.

## Teaser

KRAS variants exhibit structural and functional differences affecting their stability and interactions with other proteins.

## INTRODUCTION

**Fig. 1.**
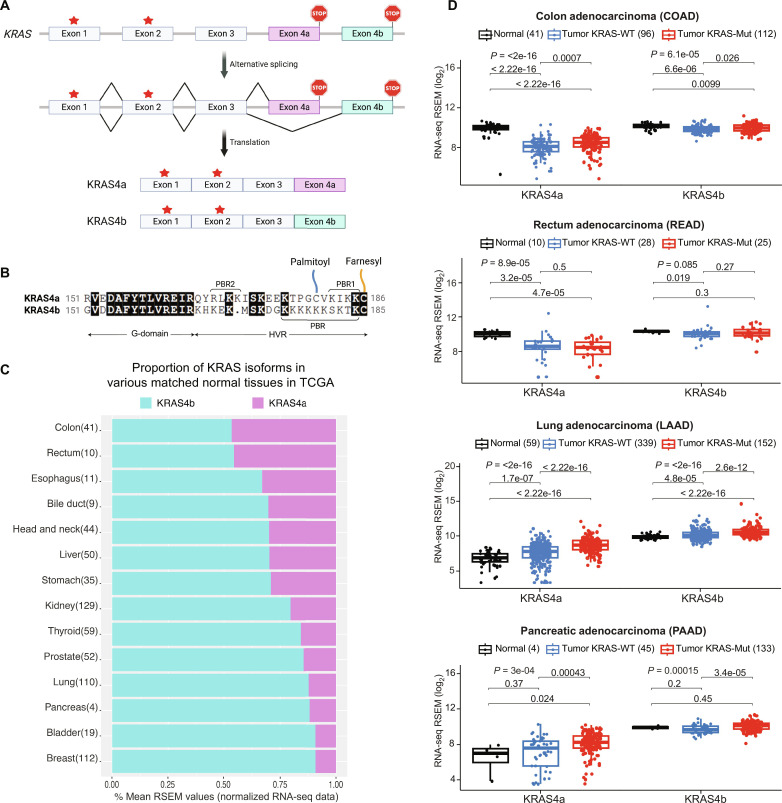
Alternative splicing of KRAS and transcript levels of the KRAS isoforms in matched normal and tumor tissues. (**A**) Alternative splicing of the *KRAS* gene results in two different isoforms, KRAS4a and KRAS4b. The red stars indicate oncogenic hotspot mutations in exons 1 and 2, part of both splice variants. (**B**) Sequence alignment of amino acid residues encoded by exon 4 in KRAS4a and KRAS4b. Conserved residues are highlighted in black. Posttranslational modifications and PBRs are indicated. (**C**) A percent stacked bar chart showing the mean transcript abundance of KRAS isoforms 4a and 4b across different matched normal tissues in the TCGA database. This analysis used the mean RSEM values of RNA-seq data for the two isoforms at the transcript level. Each bar is labeled with a specific organ or tissue, and the value in parentheses indicates the number of matched normal samples available for that tissue type in the database. The bar graph is arranged by KRAS4a transcript abundance from high (top) to low (bottom). (**D**) Comparison of the transcript abundance of KRAS4a and KRAS4b in three different sample types—matched normal tissue, tumors expressing WT KRAS, and tumors expressing mutant KRAS—in colon (COAD), rectum (READ), lung (LUAD), and pancreatic (PAAD) tissues present in the TCGA database. Transcript levels of KRAS4a and KRAS4b for individual samples from the three sample types are plotted as boxplots, with individual data points shown for each sample. *P* values shown at the top of each subgroup are derived from a nonparametric Kruskal-Wallis one-way analysis of variance test among the three subgroups of sample types. Individual pairwise tests for significant changes are also labeled.

Approximately 20% of human cancers arise because of mutations in RAS proteins. Among the three primary *RAS* genes found in mammals (*KRAS*, *NRAS*, and *HRAS*), *KRAS* has a distinct characteristic; it undergoes alternative splicing, resulting in two different splice variants (*1*). These variants, known as KRAS4a and KRAS4b, differ in their utilization of alternative fourth exons (Fig. 1A). Mutations in KRAS account for 86% of RAS-related cancers, including pancreatic, colorectal, and lung cancers. Hotspot mutations that activate KRAS occur in exons 1 and 2; consequently, both splice variants encode oncogenic proteins when expressed from a mutated allele. Despite this fact, the focus of KRAS research over four decades has been primarily on KRAS4b. Because of its comparatively lower expression levels, KRAS4a was often regarded as the “minor” splice variant and was largely ignored (*1*). However, we now know that *KRAS4a* was the transforming gene within the Kirsten murine sarcoma virus (*2*), which led to the discovery and subsequent naming of *KRAS*. Recent studies have shown the expression of KRAS4a in cancer and have elucidated differential roles for both KRAS4a and KRAS4b (*1*, *3*–*11*). As a result, there is a growing need to comprehensively understand the biochemical, structural, and functional similarities and differences between these two isoforms of KRAS.

RAS proteins act as binary switches, transitioning between the active [guanosine triphosphate (GTP)–bound] and inactive [guanosine diphosphate (GDP)–bound] forms. Structural analysis of the active and inactive RAS forms revealed similarities in their tertiary structures, except for the switch I and switch II regions. Guanine nucleotide exchange factors (GEFs) facilitate the transition from the inactive to the active state by catalyzing GDP-to-GTP exchange. Guanosine triphosphatase (GTPase)–activating proteins (GAPs), such as NF1 and RASA1/p120GAP, reverse RAS activation by increasing the GTP hydrolysis rate. In its active state, RAS interacts with the RAF kinases, phosphatidylinositol 3-kinase (PI3K), and RALGDS to initiate downstream signaling pathways. RAS isoforms share a highly similar GTPase domain (G-domain; residues 1 to 166) but exhibit notable variation in the hypervariable region (HVR) comprising the last 20 amino acids, which is crucial for RAS anchoring to the plasma membrane (PM). This anchoring is necessary for active RAS signaling and occurs through specific lipid modifications of the HVR (*12*). All RAS proteins are farnesylated at the C-terminal CaaX motif (C = cysteine; a = aliphatic amino acid; X = any amino acid). KRAS4a, like HRAS and NRAS, also undergoes palmitoylation at a cysteine residue upstream of the CaaX motif. In contrast, KRAS4b lacks palmitoylation sites and instead relies on a polybasic region (PBR) containing lysine residues that interact with lipid headgroups to bind to the membrane (*13*). HRAS, NRAS, and KRAS4a share a conserved short PBR (PBR1) in the HVR. In addition, KRAS4a has a second polybasic sequence (PBR2) between the palmitate and farnesyl modifications, which facilitates PM targeting in the absence of palmitoylation (Fig. 1B) (*9*). Maximal signaling efficiency requires both palmitoylation and PBRs. Disruption of these regions or loss of palmitoylation reduces extracellular signal–regulated kinase (ERK) phosphorylation and eliminates KRAS4a’s leukemia-inducing ability in mice (*9*).

The alternative fourth exons in the splice variants *KRAS4a* and *KRAS4b* encode the C-terminal helix α5 of the G-domain and the HVR involved in localization to the membrane. The distinct membrane-anchoring mechanisms used by KRAS4a and KRAS4b indicate different dynamics in their association with the cell membrane. Recent investigations have revealed isoform-specific interactions between KRAS4a and the RAS effectors Sin1 (*4*) and hexokinase I (*3*). These isoform-specific interactions are likely due to the localization of KRAS4a and KRAS4b to discrete membrane environments mediated by their unique HVRs. Evidence of KRAS isoform distinctiveness is mounting at the level of both transcription and protein expression. Tsai *et al.* (*9*) used splice junction priming to develop a reverse transcription polymerase chain reaction (RT-PCR) assay to quantify both KRAS transcripts. KRAS4a was found to be expressed in all 30 cell lines that were examined. The expression levels of KRAS4a were comparable to those of KRAS4b over a set of 17 fresh colorectal tumor samples. In a subsequent study, the gene expression profiles of different RAS isoforms were measured in a comprehensive panel of mouse tissues, encompassing the developmental timeline from embryogenesis to adulthood (*7*). This approach revealed a hierarchical pattern of relative contributions to total RAS expression, with KRAS4b being predominant, followed by NRAS and KRAS4a, whereas HRAS had a comparatively low contribution. Notably, the authors found that KRAS4a displayed the most dynamic regulation among the RAS isoforms, with significant up-regulation observed in the stomach, intestine, kidney, and heart during the preterm stage (*7*).

A recent study has shown that KRAS4a and KRAS4b have been conserved over more than 400 million years of evolution, which naturally raises the question as to what distinct biological roles they play and how their sequence differences enable these roles (*14*, *15*). Voice *et al.* (*16*) demonstrated that hotspot mutations at codons 12 and 13 in exon 2 and codon 61 in exon 3 lead to constitutively active KRAS4a and KRAS4b proteins, with each protein being able to transform cells. Moreover, this study revealed that oncogenic KRAS4a had a more substantial impact on forming transformed foci and enabled greater anchorage-independent growth than oncogenic KRAS4b, despite equivalent expression levels (*16*). Recently, Salmón *et al.* (*8*) generated a genetically engineered *KRAS4b*-null mouse by selectively disrupting the expression of the KRAS4b isoform. The absence of KRAS4b did not disrupt gestation but did result in mortality at or shortly after birth, highlighting the essential role of endogenous KRAS4b in mouse development (*8*). Furthermore, this study demonstrated that the expression of endogenous KRAS4a-G12V in the absence of KRAS4b was sufficient to induce lung adenocarcinomas.

Owing to its prominence as the most frequently mutated gene in human cancers, *KRAS* has emerged as a central focus in drug discovery efforts (*17*–*21*), with current strategies targeting both splice variants. Consequently, it is imperative to comprehend the structural, biochemical, and cellular attributes of KRAS4a and KRAS4b to identify potential vulnerabilities that can be leveraged for drug discovery. A comprehensive understanding of KRAS4a and KRAS4b is also vital for determining how differential localization leads to isoform-specific interaction with downstream effectors and activation of signaling pathways. In this study, we used a multifaceted approach using in vitro and cell-based assays, crystallography, nuclear magnetic resonance (NMR), and computational analysis to investigate the similarities and differences between KRAS4a and KRAS4b. Our findings revealed distinct thermal stability profiles for KRAS splice variants and identified the residues contributing to the observed differences. We present the structures of both the active and inactive forms of KRAS4a, as well as of the complex between KRAS4a and the primary effector protein RAF1 and compare them to previously reported KRAS4b structures. The NMR analysis confirmed that the backbone structural differences between the KRAS splice variants are localized in the neighborhood of their sequence variations and provided a solution-state conformational ensemble. Furthermore, experiments involving full-length KRAS variants or prenylated HVR revealed distinct functional differences, highlighting the significance of HVR variations in cellular localization and interaction with trafficking proteins and downstream effectors like RAF and PI3K within the cell.

## RESULTS

### Expression levels of two KRAS isoforms in normal and tumor tissues

To assess the expression levels of the KRAS4a and KRAS4b isoforms, we conducted an analysis of data extracted from The Cancer Genome Atlas (TCGA). Specifically, we examined the RNA sequencing (RNA-seq) data at the transcript levels of gene isoforms and the genome-wide mutation data for the mutation status of *KRAS*. Our analyses showed that in certain normal tissues such as the colon and rectum, the transcript abundance of *KRAS4a* closely matches that of *KRAS4b* (Fig. 1C). Furthermore, in the normal tissues of the esophagus, bile duct, liver, and stomach, the transcript levels of *KRAS4a* constitute more than 30% of the total *KRAS* transcripts. We note that for certain tissues (rectum, esophagus, bile duct, and pancreas) there are only a small number of matched samples currently available in TCGA, and thus, the calculated *KRAS4a*:*KRAS4b* proportion could change as additional matched samples are added to the database.

The transition from normal tissue to cancerous tissue seems to have a more pronounced impact on the transcription of *KRAS4a* compared to *KRAS4b*, particularly in colon, rectal, lung, and pancreatic cancers. Figure 1D illustrates the transcription levels of *KRAS4a* and *KRAS4b* in normal tissues, tumors expressing wild-type (WT) KRAS, and tumors expressing mutant KRAS. In colon tumors, there is a statistically significant reduction in transcription of both *KRAS4a* and *KRAS4b* compared to levels in normal tissues, regardless of *KRAS* mutation status. In rectal cancer, *KRAS4a* transcription is again significantly reduced in tumors regardless of whether they express WT or mutant KRAS; *KRAS4b* transcript abundance is lower in tumors expressing WT KRAS but not significantly lower in tumors expressing mutant KRAS. On the other hand, in lung tumors, the transcriptional abundance of both *KRAS4a* and *KRAS4b* is significantly increased compared to their abundance in normal tissues, with greater increases observed for tumors expressing mutant KRAS relative to tumors expressing WT KRAS. Last, in pancreatic tumors expressing mutant KRAS4a, a statistically significant increase in transcript abundance was observed, but not in tumors expressing WT KRAS4a. Compared to transcript levels in normal pancreatic tissue, no statistically significant changes in *KRAS4b* transcript levels were observed in pancreatic tumors. We note that the analysis of the pancreatic data is limited by the small number of data points (four) available for normal pancreatic tissue in TCGA. The causes and purposes of isoform-specific alterations in KRAS transcriptional abundance during the transition to cancer are currently unknown, underscoring the distinct but only partially understood roles played by KRAS4a and KRAS4b in specific physiological contexts.

### Biophysical and biochemical analysis of KRAS4a

**Fig. 2.**
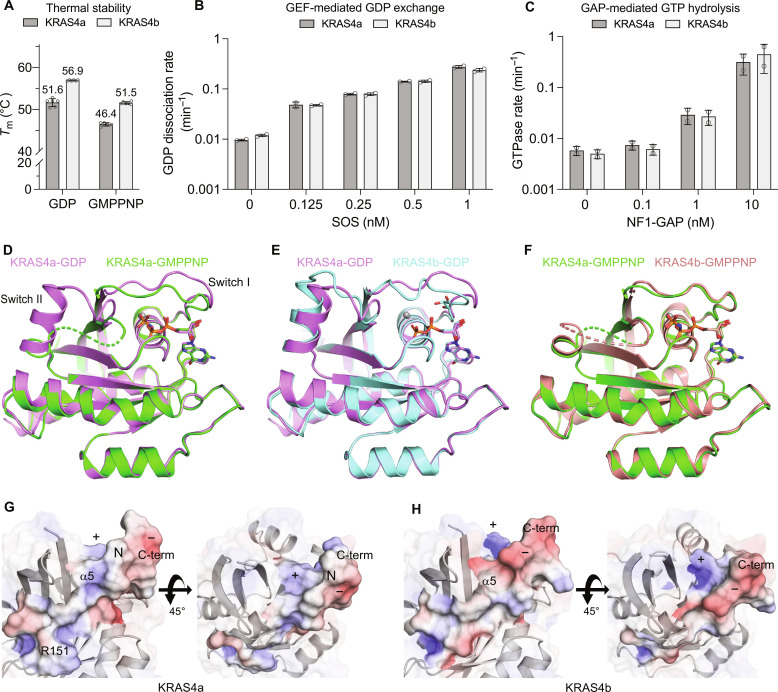
Biochemical and structural characterization of KRAS4a. (**A**) Comparison of melting temperatures (*T*_m_) for KRAS4a and KRAS4b in the active (GMPPNP-bound) and inactive (GDP-bound) states. The empty circles represent the individual data points, and the error bars represent the SD calculated from multiple replicates. (**B**) Comparison of intrinsic and SOS1 (GEF)–stimulated GDP release by KRAS4a and KRAS4b. (**C**) Comparison of intrinsic and NF1 (GAP)–mediated GTP hydrolysis by KRAS4a and KRAS4b. (**D**) Structural superpositions of KRAS4a in active (green) and inactive (pink) states. (**E** and **F**) Structural superpositions of KRAS4a and KRAS4b in the inactive (E) and active (F) states. In all structural figures, the bound nucleotide is shown as sticks, and magnesium ions are shown as gray spheres. (**G** and **H**) Electrostatic surface around the α5 helix of KRAS4a-GDP (G) and KRAS4b-GDP (H). The surface of residues 151-end is opaque, while the rest of the surface is transparent. The symbols +, N, and − indicate areas of positive, neutral, and negative potential, respectively. The spatial orientation of the proteins is the same in both panels.

We expressed and purified both KRAS isoforms and characterized their thermal stability by measuring their melting temperature (*T*_m_) using differential scanning fluorimetry (DSF). Results from these experiments revealed that KRAS4a (1 to 169) has a substantially lower (approximately 5°C) melting temperature than KRAS4b (1 to 169) in both the GDP- and Guanosine 5′-[β,γ-imido]triphosphate (GMPPNP)-bound (a nonhydrolyzable analog of GTP) forms (Fig. 2A and fig. S1A). Furthermore, both isoforms exhibited lower thermostability when bound to GMPPNP than when bound to GDP. Next, we investigated whether the amino acid differences causing different thermal stability resulted in any biochemical differences between the two isoforms. As part of their normal function, RAS proteins cycle between an inactive GDP-bound state and an active, signaling-competent GTP-bound state. We assayed the isoforms’ intrinsic ability to release GDP and their ability to release GDP when stimulated by the GEF, SOS1 (Fig. 2B and fig. S1B). In both the absence and presence of SOS1, the GDP dissociation rates were similar for the two KRAS variants. We then measured the intrinsic and NF1 GAP-mediated GTP hydrolysis rates for both KRAS isoforms (Fig. 2C and fig. S1C). The results showed that the GTP hydrolysis rates of both isoforms were similar in the absence and presence of the GAP NF1. These results suggest that despite their distinct thermal stabilities due to amino acid differences in exon 4, KRAS4a and KRAS4b exhibit comparable intrinsic activities and are similarly responsive to GEF- or GAP-mediated modulation.

### Crystal structures of the active and inactive forms of KRAS4a

To gain structural insights into the active and inactive states of KRAS4a, we solved the crystal structure of both inactive (GDP-bound) and active (GMPPNP-bound) KRAS4a at 1.8 and 1.6 Å, respectively (Table 1). Although the longest KRAS4a constructs that yielded crystals were from residues 1 to 177, interpretable electron density typically ended around residue 169 or 170. There are six amino acids that differ between KRAS4a and KRAS4b within residues 1 and 169. These six differing residues are located on helix α5, with four belonging to the G-domain and the remaining two located within the HVR (Fig. 1B). Figure 2D shows the superposition of active and inactive KRAS4a. The overall structural similarity is high, especially outside of the switch regions; the two structures have a backbone root mean square deviation (RMSD) of 0.74 Å over all residues present in both structures. The largest structural dissimilarities were found in switch I (residues 30 to 38), which is visible in both structures, and switch II (residues 60 to 76), which was disordered from residues 61 to 70 in the active, GMPPNP-bound form. Unlike GDP-bound KRAS4a, the switch I region in GMPPNP-bound KRAS4a is in a conformation compatible with its binding to downstream effectors. Figure 2 (E and F) shows a superposition of the KRAS4a structures solved in the present work and the existing structures of KRAS4b-GDP [Protein Data Bank (PDB) 6MBU] and KRAS4b-GMPPNP (PDB 6VC8), respectively. In the GDP-bound state, the KRAS4a and KRAS4b structures have an RMSD of 0.87 Å, and, again, structural differences are primarily found in switches I and II. In the GMPPNP-bound form, the KRAS4a and KRAS4b structures have higher similarity, having an RMSD of only 0.37 Å due to similar conformations of switch I for effector binding. For both GMPPNP-bound isoforms, there was no interpretable electron density for switch II from residues 61 to 70, suggesting higher flexibility of this region in the active conformation.

**Table 1.** Crystallographic data collection and refinement statistics.

	KRAS4a(1–177)-GDP	KRAS4a(1–177)-GMPPNP	KRAS4a(1–169)-R151G-GDP	KRAS4a(1–177)-GMPPNP: RAF1(RBD)	KRAS4a(1–177)-GMPPNP: RAF1(RBD-CRD)
Data collection					
Wavelength (Å)	0.9791	0.9791	0.9791	0.9791	0.9791
Resolution range (Å)	40.82–1.8 (1.864–1.8)*	44.5–1.6 (1.657–1.6)	42.39–1.5 (1.554–1.5)	38.2–1.65 (1.709–1.65)	46.45–2.65 (2.747–2.65)
Space group	*C*2	*P*2_1_	*P*3	*P*2_1_	*C*2
Unit cell					
Lengths (Å)	132.9, 34.5, 94.8	43.9, 102.1, 54.0	84.8, 84.8, 41.7	53.4, 37.7, 70.9	104.4, 199.5 113.4
Angles (°)	90.0, 120.6, 90.0	90.0, 113.8, 90.0	90.0, 90.0, 120.0	90.0, 101.7, 90.0	90.0, 112.3, 90.0
Total reflections	231,753 (23,218)	286,748 (27,396)	342,745 (20,064)	103,781 (5,725)	199,479 (20,376)
Unique reflections	34,102 (3,391)	56,122 (5,540)	52,405 (4,293)	31,825 (2,403)	61,032 (6,070)
Multiplicity	6.8 (6.8)	5.1 (4.9)	6.5 (4.7)	3.3 (2.4)	3.3 (3.4)
Completeness (%)	97.5 (97.9)	97.9 (97.1)	97.5 (79.4)	94.6 (71.9)	98.4 (97.7)
Mean *I*/sigma(I)	11.6 (1.2)	11.6 (1.2)	18.0 (3.1)	15.4 (1.5)	10.7 (1.2)
Wilson *B*-factor	27.5	27.9	18.2	25.2	63.0
*R*-merge	0.111 (1.871)	0.062 (0.995)	0.062 (0.489)	0.043 (0.610)	0.082 (0.959)
*R*-meas	0.120 (2.024)	0.069 (1.113)	0.067 (0.549)	0.051 (0.797)	0.098 (1.139)
*R*-pim	0.046 (0.763)	0.029 (0.490)	0.025 (0.242)	0.028 (0.503)	0.053 (0.609)
CC1/2	0.998 (0.503)	0.998 (0.661)	0.999 (0.886)	0.999 (0.650)	0.997 (0.501)
CC*	1.0 (0.818)	1.0 (0.892)	1.0 (0.969)	1.0 (0.888)	0.999 (0.817)
Refinement					
Reflections used in refinement	34,084 (3,381)	56,094 (5,524)	52,397 (4,291)	31,812 (2,400)	61,014 (6,063)
Reflections used for *R*-free	1,704 (169)	2,806 (277)	2,620 (215)	1,511 (114)	2,013 (200)
*R*-work	0.180 (0.255)	0.200 (0.307)	0.155 (0.198)	0.174 (0.388)	0.177 (0.334)
*R*-free	0.213 (0.306)	0.238 (0.329)	0.189 (0.236)	0.192 (0.429)	0.217 (0.358)
Number of non-hydrogen atoms	2,969	3,980	3,043	2,174	9,856
Macromolecules	2,754	3,716	2,637	1,942	9,639
Ligands	66	100	58	33	140
Solvent	149	164	348	199	77
Protein residues	339	463	326	238	1,196
RMS bonds (Å)	0.011	0.025	0.01	0.008	0.004
RMS angles (°)	1.1	1.92	1.14	1.01	0.49
Ramachandran favored (%)	98.5	97.5	98.1	98.7	96.6
Ramachandran allowed (%)	1.5	2.5	1.9	1.3	3.4
Ramachandran outliers (%)	0	0	0	0	0
Rotamer outliers (%)	0.66	0.97	0.34	0.46	0.85
Clash score	2.7	5.3	3.4	2	3.4
Average *B*-factor	35	45.2	26.9	38.1	75.8
Macromolecules	34.8	45.6	25.9	38.2	76
Ligands	44.2	36.1	15.0	23.9	65.2
Solvent	42.1	43.1	35.9	39.8	61.7

*Values in parentheses are for the highest-resolution shell.

Since helix α5 contains all six amino acids that differ between KRAS4a and KRAS4b within residues 1 to 169, we examined the electrostatics of the α5 region in both crystal structures (Fig. 2, G and H). Notably, G151 in KRAS4b eliminated the positive surface potential of R151 in KRAS4a, exposing a narrow region with a negative potential at the start of the α5 helix. Furthermore, notable differences in surface electrostatics were observed at the helix’s C terminus, which transitions into the HVR. In KRAS4a, a distinct cluster of positive potential (R167 and K170) is separated from a slightly negative potential region by a narrow, uncharged strip, indicated in Fig. 2 (G and H) by the symbols +, −, and N, respectively. The sequence differences in KRAS4b, however, yield an expanded patch of negative potential next to the patch of positive potential without any intervening neutral surface.

### Structural analysis of sequence differences between KRAS4a and KRAS4b

**Fig. 3.**
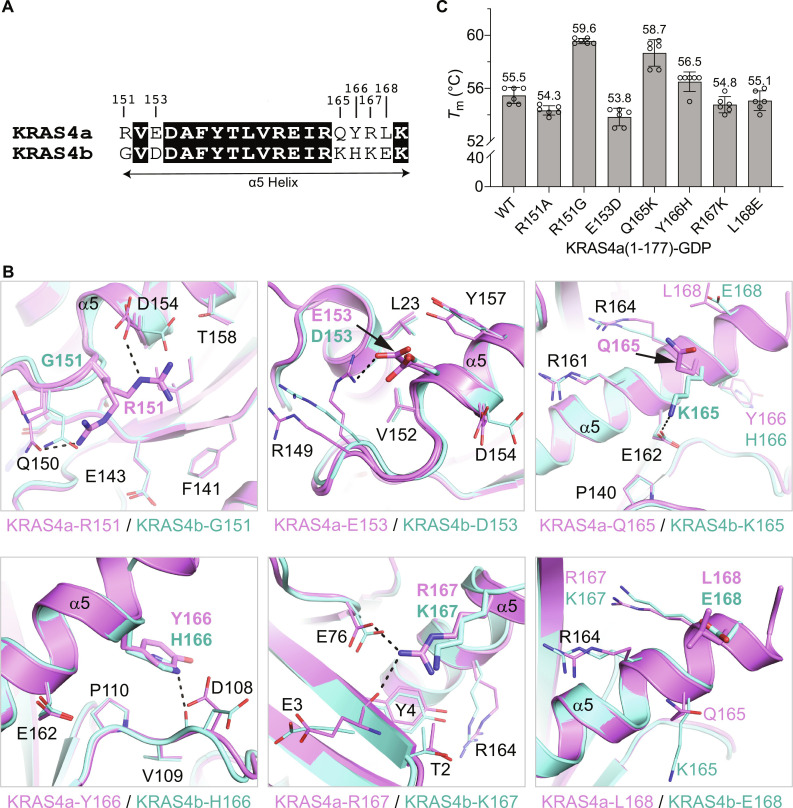
Biochemical and structural analysis of the six residues on helix α5 that differ between KRAS4a and KRAS4b. (**A**) Sequence alignment showing the position of the six sequence differences located on helix α5 between KRAS4a and KRAS4b. (**B**) Structural views highlighting the interactions formed by the six residues differing between KRAS4a and KRAS4b in the α5 helix. In each view of the six different sequence positions, the side chains located at that specific sequence position are depicted as sticks, whereas neighboring side chains are drawn as thin lines. Hydrogen bonds are shown as dashed black lines. Multiple pink side chains present at a single position indicate different conformations of that side chain in different copies of the protein in the crystallographic asymmetric unit. (**C**) Melting temperature (*T*_m_) analysis of point mutants of KRAS4a(1–177)-GDP in which the KRAS4a residue has been mutated to the corresponding KRAS4b residue. The empty circles represent the individual data points, and the error bars represent the SD calculated from multiple replicates.

Next, we compared the specific interactions between the six amino acids that differ between KRAS4a and KRAS4b within residues 1 to 169 and neighboring residues (Fig. 3A). Altered side-chain interactions resulting from amino acid variations could account for the discrepancy in thermal stability between the two KRAS isoforms. Figure 3B provides views of the structure near each amino acid difference, with KRAS4a-GDP represented in pink and KRAS4b-GDP in cyan. At position 151, the arginine residue in KRAS4a forms hydrogen bonds with neighboring residues (D154 in chain A or Q150 in chain B). However, these interactions are not possible in KRAS4b due to the presence of a glycine residue at this position. At position 153, glutamate of KRAS4a interacts with the guanidine group of the neighboring R149 in one of the two KRAS4a molecules within the asymmetric unit. This interaction is absent in KRAS4b, likely due to the shorter side chain of aspartate at position 153. Conversely, at positions 165 and 166, KRAS4b exhibits interactions that are not present in KRAS4a. In KRAS4b, K165 interacts with D162, whereas Q165 in KRAS4a points away from it. Furthermore, at position 166, a nitrogen atom from the imidazole ring of histidine in KRAS4b forms a backbone hydrogen bond with the carbonyl of D108. This interaction cannot occur with the tyrosine ring at the same position in KRAS4a. At position 167, the longer arginine side chain in KRAS4a enables it to form a salt bridge with the side chain of E76 and a hydrogen bond with the backbone carbonyl of E3. On the other hand, the slightly shorter lysine side chain in KRAS4b does not come into interaction distance with either of these residues. The final difference within residues 1 to 169 occurs at position 168. The nonpolar leucine residue in KRAS4a differs chemically from the negatively charged glutamate residue in KRAS4b. However, in both structures, the side chain of residues at this position extends away from the rest of the protein and does not exhibit observable interactions with other residues. In summary, among the six amino acid differences within residues 1 to 169 in KRAS4a and KRAS4b, three positions exhibit side-chain interactions exclusive to KRAS4a, two positions show interactions unique to KRAS4b, and one position lacks side-chain interactions in both isoforms. While the contribution of each interaction to the overall thermal stability of the protein may vary, no discernible pattern of gained or lost interactions at these six positions can readily explain the observed difference in the melting temperature between the two isoforms.

To further investigate the impact of these six residues on the thermal stability of KRAS isoforms, we generated recombinant KRAS4a single mutants in which each of the six differing residues in KRAS4a was replaced with the corresponding residue from KRAS4b: R151G, E153D, Q165K, Y166H, R167K, and L168E. We measured the thermal stability of these KRAS4a mutants using DSF, as depicted in Fig. 3C. The KRAS4a-R151G mutant exhibited the largest change in melting temperature (*T*_m_), followed by the KRAS4a-Q165K mutant. The remaining four mutants displayed only modest alterations in *T*_m_ compared to WT KRAS4a. These findings suggest that residue 151, the first amino acid in the fourth exon, and residue 165, located near the end of helix α5 adjacent to the HVR, are mainly responsible for the difference in thermal stability between the two KRAS isoforms.

### KRAS4a-R151G mutant similarity to KRAS4b

**Fig. 4.**
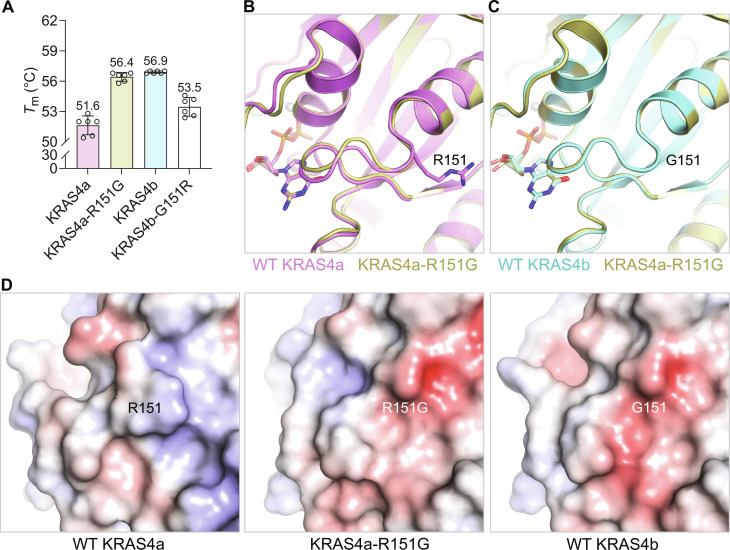
Biophysical and structural characterization of position 151 in KRAS4a and KRAS4b. (**A**) Thermal melting analysis of arginine versus glycine at position 151 in KRAS4a and KRAS4b in the GDP-bound state. The empty circles represent the individual data points, and the error bars represent the SD calculated from multiple replicates. (**B**) Structural superposition of GDP-bound WT KRAS4a and KRAS4a-R151G. (**C**) Structural superposition of GDP-bound WT KRAS4b and KRAS4a-R151G. Structures in (B) and (C) are color-coded according to (A). (**D**) Electrostatic surface representation showing enlarged view around position 151 in GDP-bound WT KRAS4a, KRAS4a-R151G, and WT KRAS4b. The spatial orientation of the protein is identical in all three depictions.

Considering the substantial influence of residue 151 on the thermal stability of both KRAS isoforms, we conducted a comprehensive analysis at this position. In-depth thermal melting experiments were performed using KRAS constructs spanning residues 1 to 169. The results demonstrated that introducing the R151G mutation in KRAS4a led to a melting temperature increase by nearly 5°C, matching the melting temperature of WT KRAS4b (Fig. 4A and fig. S2). To further investigate the impact of residue 151 on KRAS stability, we created the reverse mutation in KRAS4b by substituting its native glycine at position 151 with the arginine present in KRAS4a. The inclusion of arginine at position 151 in KRAS4b decreased its melting temperature by 4° to 55.4°C, nearly matching that of the WT KRAS4a (54.5°C). These results suggest that the amino acid at position 151 is the primary determinant of the thermal stability difference observed between KRAS4a and KRAS4b.

Next, we solved the crystal structure of the GDP-bound KRAS4a-R151G mutant and compared it with those of WT KRAS4a and KRAS4b (Table 1 and Fig. 4, B and C). In contrast to WT KRAS4a, which crystallized in the monoclinic space group *C*2, the crystals of the KRAS4a-R151G mutant exhibited the same space group, *P*3, and unit cell dimensions that we previously observed for the structure of WT KRAS4b-GDP (PDB 6MBU). This suggests that the crystallization behavior of the KRAS4a-R151G mutant is more similar to WT KRAS4b than to WT KRAS4a. Comparative analysis of the structures indicates that, while the overall structures of WT KRAS4a, KRAS4a-R151G, and WT KRAS4b are very similar to each other, the loop leading to residue 151 and helix α5 in KRAS4a-R151G is nearly identical to WT KRAS4b and less similar to WT KRAS4a. This further highlights the amino acid at position 151 as the key difference between the two KRAS isoforms. Solution NMR data also support this conclusion. Figure S3 shows the results of ^1^H-^15^N chemical shift perturbation analysis of KRAS4a-R151G versus WT KRAS4a and WT KRAS4b. Compared to WT KRAS4a, there are a small number of perturbed chemical shifts centered at position 151 in KRAS4a-R151G, a phenomenon frequently observed around the mutation sites (fig. S3A). In contrast, there are few substantial chemical shift differences in the R151G mutant compared to WT KRAS4b, which shares glycine at position 151 (fig. S3B). The large chemical shift differences between WT KRAS4a and WT KRAS4b in the vicinity of residue 166 (fig. S4B) are preserved in KRAS4a-R151G, suggesting that although the R151G mutation in KRAS4a restores most of the structural similarity to KRAS4b (spanning residues 149 to 155), the C-terminal region (i.e., residues 165 to 169) remains similar to KRAS4a (fig. S3B versus fig. S4B). Overall, the structure of KRAS4a-R151G is more similar to WT KRAS4b than to WT KRAS4a, in both the crystal and solution states, despite the fact that WT KRAS4a and the R151G mutant differ only at that single position, whereas KRAS4a-R151G and WT KRAS4b still differ at five other positions in helix α5.

Last, we analyzed the impact of the R151G mutation on the surface electrostatics of KRAS4a. The positive charge of R151 in WT KRAS4a is shown as a blue area in Fig. 4D. The absence of a long positively charged side chain in KRAS4a-R151G results in the exposure of a contiguous patch of negative (red) electrostatic potential. This surface closely resembles that of WT KRAS4b, which has a native glycine at position 151. These results indicate that, similar to its role in determining the protein’s thermal stability, the residue at position 151 is the primary determinant of whether the surface electrostatics of KRAS near the N terminus of helix α5 are more KRAS4a-like or KRAS4b-like, regardless of the other amino acids in the sequence.

### Slowly exchanging conformational states in KRAS4a-GDP from NMR chemical shifts

To understand the structural and dynamics differences that may arise because of the different melting temperatures of KRAS4a and KRAS4b, we decided to study the two KRAS isoforms using solution-state NMR. NMR was used to provide an accurate description of the solution state conformations of RAS proteins and a description of the timescales of their structural fluctuations, which likely contribute to their functional differences (*22*, *23*). First, we compared the ^1^H-^15^N spectra of KRAS4a in GDP- and GMPPNP-bound states and observed numerous amide chemical shift differences (fig. S5A). Significant chemical shift differences between the GDP- and GMPPNP-bound states are primarily localized to the P-loop, residues bordering the highly mobile switch regions, and residues near the γ-phosphate of the GMPPNP nucleotide (fig. S5B). These chemical shift differences are likely attributable directly to the presence of the γ-phosphate group found in GMPPNP. Minor but consistent chemical shift differences span helix α3 (residues 88 to 103; fig. S5B). Thus, there appears to be no significant long-range chemical shift perturbation to sites remote from the nucleotide, a result that agrees with our solved crystal structures of KRAS4a in the two states that show virtually no conformational differences outside the switch regions (Fig. 2D).

Next, we compared KRAS4a to KRAS4b in both the GDP- and GMPPNP-bound states (figs. S4, A to C, and S6, A to C). In both the GDP- and GMPPNP-bound states, the largest chemical shift differences between KRAS4a and KRAS4b are primarily localized to helix α5, which contains all amino acids that differ in the G-domain of the two isoforms, and the loop leading to it (residues 142 to 150). Additional propagation of small chemical shift changes is present in the neighboring β5 strand and its adjacent loops (residues 109 to 119). Overall, the chemical shift differences between the two isoforms are explained by the sequence differences found on helix α5, and there is no substantial chemical shift perturbation throughout the backbone that could indicate substantial conformational differences, in agreement with the similarity of the crystal structures (Fig. 2, E and F). Using ^31^P NMR, we examined the GMPPNP-bound state of both KRAS4a and KRAS4b in more detail, as doubling of the γ-phosphate peak in the ^31^P spectrum of RAS is known to accurately report the relative populations of the major functional states of the RAS effector lobe, known as state I and state II. The γ-phosphate peaks in the ^31^P NMR spectra of KRAS4a and KRAS4b in the GMPPNP-bound state (fig. S7) are indistinguishable between the 4a and 4b isoforms, indicating that the population ratio of states I and II are similar in the two isoforms. State II is the RAS conformation competent to bind effectors, and thus, both isoforms should be equally competent to interact with effectors.

**Fig. 5.**
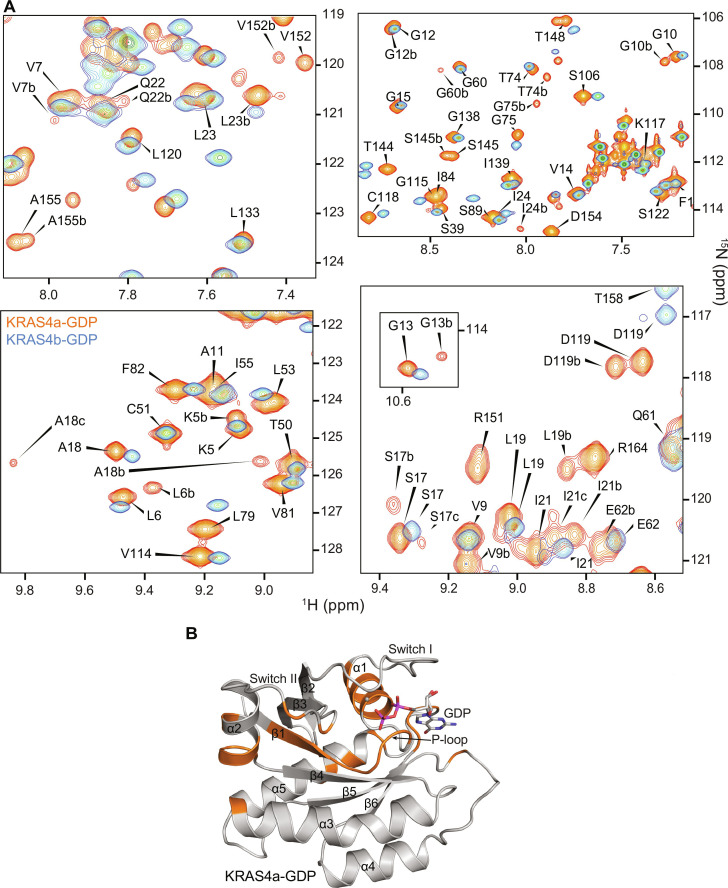
Identification of slow conformational exchange present in KRAS4a-GDP but not in KRAS4b-GDP. (**A**) Cross-peaks for certain residues in the ^1^H-^15^N HSQC spectrum of KRAS4a-GDP (in orange) indicate multiple conformations in slow exchange on the NMR timescale. Sequence assignments for the orange resonances are given in black, and residues exhibiting multiple conformations are indicated with lowercase letters following the assignment (e.g., A18, A18b, and A18c). In contrast, KRAS4b-GDP resonances (overlaid in blue) do not exhibit multiple conformations, suggesting either fast exchange or a single rigid conformation on the NMR timescale. (**B**) The KRAS4a-GDP residues exhibiting multiple conformations are mapped in orange onto the crystal structure.

While performing standard NMR backbone assignments of KRAS4a in the GDP- and GMPPNP-bound states, we noticed the presence of additional conformers slowly exchanging on the NMR timescale in KRAS4a-GDP but not in KRAS4a-GMPPNP or in KRAS4b in either nucleotide-bound state (Fig. 5A). The additional conformations manifest themselves through the presence of two or more amide peaks for certain residues. The residues with multiple amide peaks include K5-V9 (N-terminal strand β1), G10-G13 (P-loop), S17-I24 (helix α1), and G60, E62, Q70, and M72-G75 (switch II). In addition, amide peak doubling is observed for V103 (whose side chain is oriented toward switch II), D119 and S145-K147 (all having side chains oriented toward the base of GDP), and V152, A155, and T158 (all located on the C-terminal helix α5). These residues showing slow conformational exchange, which are mapped onto the three-dimensional (3D) structure (Fig. 5B), include not only those that directly interact with the mobile switch II but also regions thought to be rigid (α1, β1, P-loop, and GDP). Residue K104 was observed to regulate the oncogenic activity of KRAS mutants through an allosteric network with M72, R73, and G75 on the α2 helix of the switch II region (*24*). In GDP-bound KRAS4a, the residue V103 (adjacent to K104) and residues M72-G75 (switch II) show peak splitting, suggesting that a similar interaction mediates the corresponding conformations. In a previous study, we observed that strongly coupled (synchronized) motions connect the effector and allosteric lobes of KRAS4b in the GMPPNP-bound form, while faster and more complex motion occurs in its GDP-bound form (*22*). The corresponding slow exchanging conformations in KRAS4a-GDP suggest a possible allosteric pathway between switches I and II, mediated by β1, the P-loop, and α1 (effector lobe), with possible cross-talk with the C-terminal α5 (allosteric lobe). Residues S17-A18 and I21-Q22 exhibited a third set of peaks, which is indicative of the presence of a third slowly exchanging minor conformer. This contrasts with the GDP-bound KRAS4b spectrum, in which a corresponding additional set of slowly exchanging peaks is not visible, suggesting that the GDP-bound KRAS4b either is structurally rigid or, more likely, undergoes dynamics on a faster timescale compared to KRAS4a (*22*). Considering that all six residues that are different between the two KRAS isoforms are localized on α5, this suggests that the differences in thermal stability between the two isoforms of the G-domain are related to subtle variations in conformational dynamics. Motion originating in the highly mobile switch regions in the effector lobe is likely propagated throughout the core of the protein on a path containing the residues that show multiple amide peaks in the GDP-bound state. We note that one of the residues exhibiting peak doubling in helix α5 is V152, adjacent to residue 151, which we previously observed to have the largest effect on the thermal stability of any of the six sequence differences between the two isoforms. In KRAS4b, the presence of the more conformationally flexible glycine at position 151 compared to the arginine found in KRAS4a could alter the dynamics of helix α5 on timescales that contribute to the increased thermal stability observed for the KRAS4b isoform. It is conceivable that each of the six residues that are different between the two isoforms of the KRAS G-domain has a distinct effect on the dynamics of the protein, reflected in their relative thermal stability contributions. This is supported by the fact that the peak doubling observed in the GDP-bound KRAS4a (Fig. 5) is completely abolished by the R151G mutation, which proves that the slow conformational exchange observed for residues in Fig. 5 becomes much faster in KRAS4a-R151G. This single-point mutation therefore exhibits conformational dynamics similarity to GDP-bound KRAS4b.

### Subtle structural and dynamical differences between solution and crystal structures of the KRAS isoforms from RDCs

Because we detected noticeable chemical shift differences between the two KRAS isoforms (figs. S4 and S6), we decided to measure the residual dipolar couplings (RDCs), which are sensitive reporters of subtle differences in structure and dynamics in solution. The agreement between the average solution conformation of a molecule and a crystal structure can be assessed by fitting experimentally measured RDCs to the crystal structure. If the RMSD of the fit is noticeably larger than the precision in measuring the RDCs, a better representation of the average solution conformation can be obtained by performing a gentle structural refinement under RDC restraints so that the RDCs back-calculated from the structure match the measured values within their experimental precision. Measuring RDCs in two independent alignment media greatly improves the quality of the RDC-refined structures, and in this study, we used Pf1 filamentous phage and bicelles as independent alignment media for partial alignment of the protein molecules in solution.

For slow-exchanging residues exhibiting multiple amide peaks in the KRAS4a-GDP spectra, only the RDCs of the strongest peak (corresponding to the major conformer) were included in the analysis. When fitting the ^1^D_NH_ and ${}^{1}D_{C^{\alpha }H^{\alpha }}$ RDCs measured in Pf1 to chain A (fig. S8, A and C) and chain B (fig. S8, B and D) of the crystal structure of KRAS4a-GDP, the outliers map to T35 and the switch I and II regions (fig. S8E), indicating that the crystal structures are reasonable models for the average solution structure but not for either the major or the average conformations of the flexible switch regions in which crystal packing likely affects the backbone conformation in the crystalline state. When the outliers are excluded, the experimental RDCs agree substantially better with those back-calculated from the structure (fig. S8F). The fact that the measurement error associated with the experimental RDCs is much smaller than the RMSD values obtained by fitting those RDCs to the various crystal structures indicates that real structural differences, however subtle, exist between the isoforms’ average solution and crystal structures and suggests that refining the crystal structures against solution RDC data may provide a better representation of the average solution-state conformation. Therefore, we generated RDC-refined solution structures that fit the RDC restraints to a degree closely approaching their experimental measurement errors. Because there are only six residues that differ between the two KRAS (residues 1 to 169) isoforms, we compared the experimental Pf1 ${}^{1}D_{NH}/{}^{1}D_{C^{\alpha }H^{\alpha }}$ RDCs of KRAS4a to those of KRAS4b in both the GMPPNP-bound and GDP-bound forms (fig. S9, A to D) and noted that the RDCs of the two isoforms correlate better in the active state, which suggests that the corresponding active state structures of the G-domains of the two isoforms are quite similar in solution. The degree of correlation between the measured RDCs of the two isoforms in the GDP-bound state is lower, possibly due to the existence of multiple states with fast and distinct conformational dynamics in KRAS4b (*22*), in contrast to KRAS4a, which exhibits slowly exchanging conformers as shown in Fig. 5. Because the observed RDCs represent time-averaged measurements over a timescale of approximately 100 ms, the distinct and more complex dynamics present in the inactive, GDP-bound states of the two KRAS isoforms led us to refine by RDCs only the GMPPNP-bound active state of KRAS4a.

**Fig. 6.**
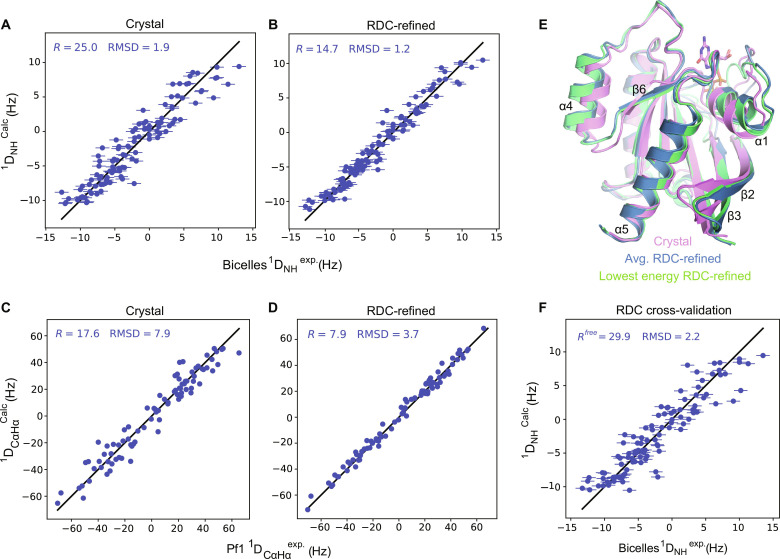
RDC-refined structures reveal subtle differences between solution and crystal forms of KRAS4a-GMPPNP. (**A** and **B**) Correlation plot of experimental NH RDCs measured in bicelles versus RDC values back-calculated from the crystal structure (A) and the lowest-energy RDC-refined structure (B). (**C** and **D**) Correlation plot of experimental C_^α^_H_^α^_ RDCs measured using Pf1 filamentous phage as the alignment medium versus RDC values back-calculated from the crystal structure (C) and the lowest-energy RDC-refined structure (D). In (A) to (D), the *R*-factor (%) is a unitless measure of the agreement between the experimental and back-calculated RDC values, with 0% indicating perfect agreement and higher values indicating progressively worse agreement; RMSD values are given in hertz. (**E**) Superposition of the KRAS4a-GMPPNP crystal and RDC-refined structures. The crystal structure used as the starting point for RDC refinement is shown in pink, the lowest-energy RDC-refined structure in green, and the average structure from the bundle of 20 RDC-refined structures in blue. Secondary structural elements that differ between the crystal and the RDC-refined structures are labeled. (**F**) A sample cross-validation plot showing the agreement between the experimental NH RDCs and values back-calculated from the structure refined without using the NH RDC data. *R*_free_ is analogous to the *R*-factor in the other plots; the subscript “free” indicates that it is calculated for data excluded from the refinement process itself.

We refined the crystal structure of KRAS4a in the GMPPNP-bound state via simulated annealing using molecular dynamics and gradient minimization under restraints derived from the ^1^D_NH_ and ${}^{1}D_{C^{\alpha }H^{\alpha }}$ data from both alignment media, carefully adjusting the force constant of each set of RDC restraints to yield structures that fit the RDCs within the experimental errors to avoid overfitting. A comparison of Fig. 6 (A and C) with Fig. 6 (B and D), respectively, reveals that the refined structures predict the observed data more accurately than the original crystal structure. Figure 6E shows a superposition of the starting crystal structure, lowest-energy RDC-refined structure, and average RDC-refined structure. The 20 lowest-energy structures refined with RDCs have an RMSD of 1.1 Å, while the RMSD between the average of these structures and the starting x-ray structural model is 2.0 Å. Overall, the RDC-refined structures are less compact than the crystal structures, with differences in the position of α1, which is connected to the highly mobile switch I, the β2-β3 hairpin loop, switch II (highly mobile), α4, and the segment from β6-α5. Some of the structural displacements relative to the crystal structure likely result from the lack of crystal packing forces on those structural elements when in solution, but others could be due to the intrinsic dynamics in solution that are damped in the crystalline state. Separate cross-validation calculations were performed by excluding each individual RDC set to calculate the *R*^free^ quality factor (*25*). Both the *R* and *R*^free^ quality factors, expressed as a percentage, measure the agreement between the experimental RDCs and those back-calculated from a 3D structure, with lower *R* or *R*^free^ values corresponding to a better agreement. All the cross-validated structures fit the RDCs similarly to, but not better than, the starting crystal structure as illustrated, for example, by comparing Fig. 6 (A to F). In optimal cases, an ensemble RDC structural refinement can reveal intermediate conformational states sampled by a protein in solution, provided the conformational exchange corresponds to a simple two-state model (*26*), which is not the case for GMPPNP-bound KRAS4a. Thus, the RDC refinement yields structures that are slightly different from the crystal structure yet equally compatible with the RDC data. The crystal structure represents a single structural snapshot of the protein; by refining that structure against solution NMR data, we obtained additional structural snapshots that provide a more complete picture of the conformational sampling that the protein experiences in solution.

### Structures of KRAS4a-RAF1 complexes and comparison to equivalent RAF1 complexes with KRAS4b and HRAS

**Fig. 7.**
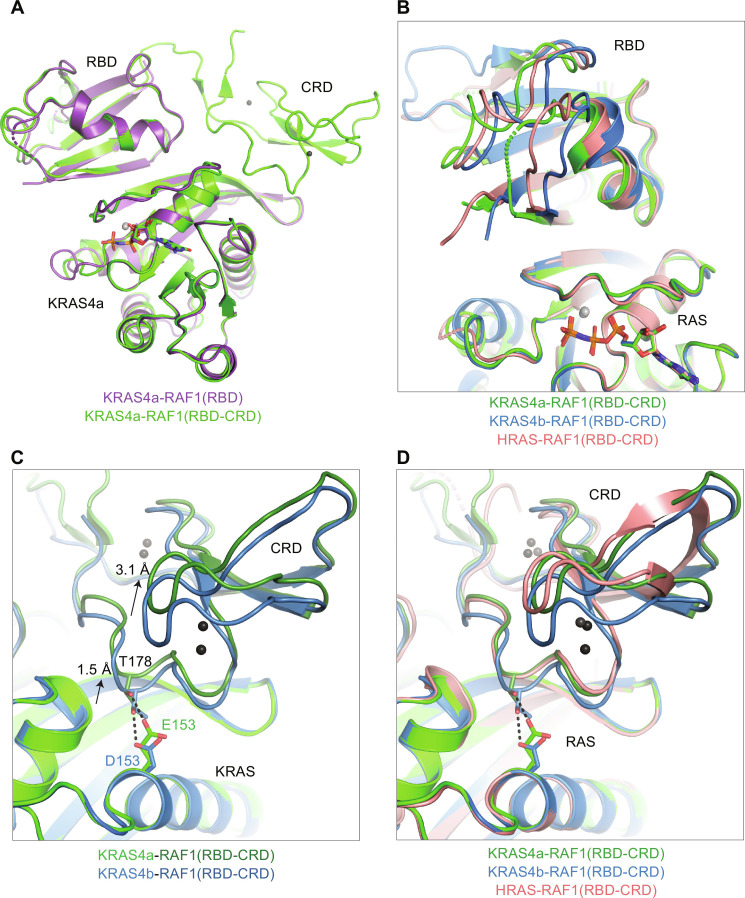
Structures of KRAS4a-GMPPNP bound to the RBD and RBD-CRD of RAF1 and structural comparison to RAF1 complexes with KRAS4b and HRAS. All structural superpositions were performed using only the RAS component of the structure. (**A**) Structural superposition of the KRAS4a:RAF1 RBD (purple) and KRAS4a:RAF1 RBD-CRD (green) structures solved in the present work. (**B**) Positional variability observed in the RAF1-RBD when bound to KRAS4a (green), KRAS4b (blue), and HRAS (salmon). (**C** and **D**) Conformational differences observed in the RAF1-CRD when bound to KRAS4a (green), KRAS4b (blue), and HRAS (salmonbeige) due to different amino acids at position 153. The longer side chain at position 153 in KRAS4a and HRAS displaces the RAF1-CRD by 1.5 to 3 Å compared to its position bound to KRAS4b. Dark gray spheres in the CRD are zinc ions.

To gain insights into the interaction between KRAS4a with its downstream effector RAF kinase, we solved the structures of KRAS4a bound to the RAS-binding domain (RBD) of RAF1 at 1.65-Å resolution and bound to the RBD and cysteine-rich domain (RBD-CRD) at 2.65-Å resolution (Table 1). Figure 7A shows the superposition of the two complexes aligned using only the KRAS4a components. Strand β2 of the RBD contacts the N-terminal section of strand β2 of KRAS4a to create an extended β sheet bridging the two proteins, whereas the RAF1 CRD interacts with KRAS4a residues located on the interswitch region and helix α5.

We compared the structure of KRAS4a:RAF1 RBD-CRD with previously reported structures of RAF1 RBD-CRD bound to KRAS4b (PDB 6XI7) and HRAS (PDB 7JHP). Figure 7B compares the position of the RBD bound to the different RAS molecules. The β strands that form the intermolecular β sheet align well structurally, but the loops and helices of the RBD show noticeable positional heterogeneity, reflecting the flexibility of the molecule away from the β sheet that anchors it to RAS. We next compared the position of the CRD when bound to KRAS4a and KRAS4b (Fig. 7C). The CRD of RAF1 interacts with position 153 on the KRAS α5 helix, one of the six residues that differ between the G-domains of KRAS4a and KRAS4b. The longer side chain of E153 in KRAS4a versus D153 in KRAS4b displaces the RAF1 CRD in complex with KRAS4a by 1.5 to 3 Å compared to the CRD’s position when bound to KRAS4b (*27*). HRAS, like KRAS4a, has glutamate at position 153, and the HRAS:RAF1 RBD-CRD complex shows a very similar displacement of the CRD to that observed in the KRAS4a complex (Fig. 7D) (*28*). These structural analyses demonstrate that the interaction of KRAS4a with RAF1 RBD-CRD is more similar to HRAS than the KRAS4b complexes with RAF1 RBD-CRD. We, therefore, propose that position 153 fine-tunes the interaction of RAS with the CRD of RAF kinase.

### Role of the HVR in KRAS4a and KRAS4b

**Fig. 8.**
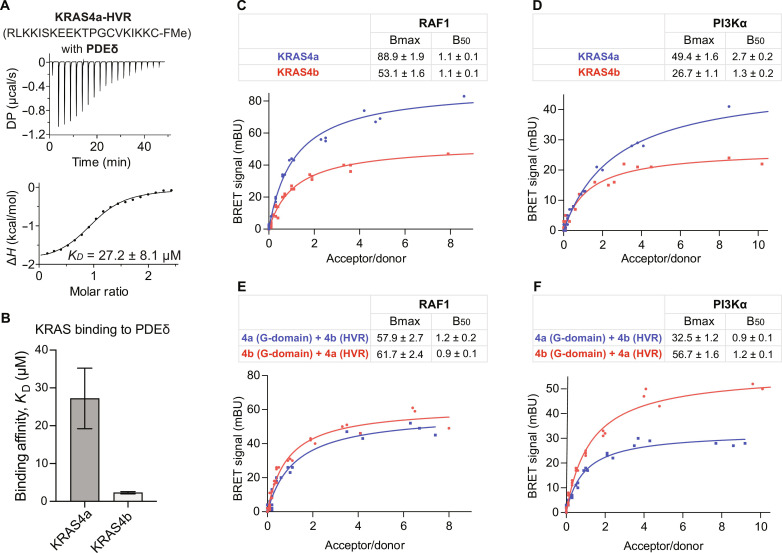
Functional differences conferred by the HVR of KRAS4a and KRAS4b. (**A**) ITC measurement of the binding affinity of PDEδ for a C-terminally farnesylated and carboxymethylated 20-mer peptide from the HVR of KRAS4a. (**B**) Bar graph comparing the binding affinity (*K*_D_) of PDEδ with KRAS4a [from (A)] and KRAS4b (*30*). (**C** and **D**) BRET saturation curve experiments between mVenus-tagged KRAS4a/KRAS4b and NanoLuc(nL)–tagged RAF1 (C) and PI3Kα (p110α) (D) constructs transiently expressed in HEK293T cells. (**E** and **F**) BRET saturation curve experiments between mVenus-tagged KRAS4a G-domain fused to the HVR of KRAS4b and vice versa with NanoLuc-tagged RAF1 (E) and PI3Kα (p110α) (F) constructs transiently expressed in HEK293T cells. NanoLuc-effector donor construct expression levels were held constant, while mVenus-RAS acceptor constructs were titrated, and adjusted BRET signal is reported as a function of acceptor/donor concentration. Data are representative of at least three biological replicates, each consisting of three technical replicates. BRET_50_ (B50) and BRET_max_ (Bmax) values from the curve fitting are shown in each panel as the fitted value ± the SE. Bmax values were found to be significantly different between KRAS4a and KRAS4b when interacting with RAF1-nL (*P* = 0.0463) and nL-p110a (*P* < 0.0001) as determined by a paired *t* test. B50 values were found to be significantly different between KRAS4b(G-domain)-KRAS4a(HVR) and KRAS4a(G-domain)-KRAS4b(HVR) when interacting with nL-p110α (*P* = 0.0449). Bmax values between these constructs were also found to differ significantly when interacting with nL-p110α (*P* = 0.0012) but not with RAF1-nL.

The regulation of KRAS4b signaling and its membrane distribution is controlled by the phosphodiesterase-δ (PDEδ) protein, which binds to prenylated proteins. Previous studies have demonstrated that PDEδ binds to KRAS4b through its prenylated HVR, facilitating its localization to membranes (*29*, *30*). Unlike KRAS4b, the HVR of KRAS4a is palmitoylated at a cysteine residue upstream of its C-terminal cysteine. This reversible palmitoylation is essential for the proper membrane localization of RAS proteins. However, PDEδ’s hydrophobic pocket cannot accommodate an additional lipid chain, which means that prenylated proteins can only bind to PDEδ when they are depalmitoylated. Previously, we showed that full-length prenylated KRAS4b binds to PDEδ with a dissociation constant (*K*_D_) of 2.3 μM, and the last five HVR residues, including prenylated Cys, interact with PDEδ to form a stable KRAS4b-PDEδ complex (*30*). To evaluate the interaction between KRAS4a and PDEδ, we measured the affinity of a prenylated 20-residue KRAS4a HVR peptide (without palmitoylation) for PDEδ using isothermal titration calorimetry (ITC). The results (Fig. 8, A and B) indicated that KRAS4a binds to PDEδ with a *K*_D_ of 27 μM, which is 10-fold weaker compared to full-length prenylated KRAS4b (*30*). This finding supports our previous observation that PDEδ may bind to depalmitoylated RAS isoforms containing residues with small side chains upstream of the prenylated cysteine (*30*). In the case of KRAS4a, the presence of two lysine residues with relatively longer side chains upstream of the prenylated cysteine likely causes steric hindrance with PDEδ residues, resulting in substantially reduced interaction between KRAS4a and PDEδ. Overall, these results suggest that PDEδ does not notably affect the localization of KRAS4a, highlighting the presence of distinct subcellular trafficking mechanisms between the two KRAS splice variants.

To understand differences in the role of the HVR between the two KRAS isoforms, we used bioluminescence resonance energy transfer (BRET) to measure the interactions of full-length KRAS4a and KRAS4b with the downstream effectors RAF1 and p110α in live cells. The system uses a bioluminescent donor tag (NanoLuc) that emits light at 460 nm after the addition of its substrate furimazine. If within 10 nm of an appropriate fluorescent acceptor tag, resonance energy transfer will occur, exciting the fluorophore which will emit light at a separate wavelength. This BRET signal, when normalized to donor readings, provides a sensitive, ratiometric readout of interactions between two proteins of interest. Individual acceptor and donor measurements also quantify the relative expression level between constructs. The tag position was optimized to obtain the highest detectable BRET signal. By maintaining the NanoLuc donor concentration at constant levels and increasing acceptor concentrations, we measured the dynamics and binding affinity between the two tagged proteins within live cells. Specific interactions saturate at a BRET_max_ value as acceptor expression increases, which reflects the maximum signal possible within the system. BRET_50_ values, analogous to *K*_D_, represent the acceptor/donor ratios necessary to reach 50% of the BRET_max_ and provide a measurement of relative affinity.

Saturation curves between mVenus-KRAS4a, mVenus-KRAS4b, and RAF1-NanoLuc revealed a significant difference in BRET_max_ values across the three biological replicates, with KRAS4a emitting a higher maximum adjusted BRET signal (Fig. 8C, *P* = 0.0463). The same was observed for NanoLuc-p110α (Fig. 8D, *P* < 0.0001). The fitted BRET parameters for all biological replicates are shown in table S1, and a statistical analysis is reported in table S2. These observations align with a previous interactome analysis study that proposed a higher affinity interaction between KRAS4a and RAF1 than KRAS4b, resulting in an enhanced RAF1–mitogen-activated protein kinase kinase (MEK)–ERK signaling cascade (*10*). To examine whether this difference between the two KRAS isoforms is due to differences in the G-domain or HVR, we created chimeric constructs of two KRAS isoforms in which the HVRs of KRAS4b and KRAS4a (residues 168 to 188/189) were swapped. When we used these chimeric KRAS constructs in the BRET assay, the BRET_max_ difference became insignificant with RAF1 (Fig. 8E) and was reversed with p110α (Fig. 8F, *P* = 0.0012). The BRET_50_ values for the chimeric constructs with p110α also showed a statistically significant difference across the four biological replicates (*P* = 0.0449), with the KRAS4a(G)-KRAS4b(HVR) construct presenting a lower BRET_50_ value and, therefore, a higher relative binding affinity. The ITC binding assay showed that the G-domains of KRAS4a and KRAS4b have comparable affinities for binding to RAF1 and PI3K (fig. S10). However, the higher BRET_max_ value of KRAS4a HVR suggests that there is a greater presence of KRAS4a on the membrane, ready to bind to downstream effectors.

Differences in the G-domain residues between KRAS4a and KRAS4b may affect their interaction with downstream effectors at the membrane, as indicated by the incomplete reversal of the BRET difference in chimeric constructs. Previous data from our melting analysis and crystal structures highlighted the importance of position 151 on the melting temperature of both KRAS isoforms and the role of position 153 in KRAS’s interaction with RAF1. Therefore, we conducted cellular BRET saturation curve assays to explore the consequences of substitutions at these positions, using four biological replicates for each substitution (table S1). BRET experiments with the KRAS4a-R151G mutant revealed statistically insignificant changes in both BRET_max_ and BRET_50_ (fig. S12A and table S2). This suggests that although position 151 significantly influences KRAS’s thermal stability, it does not measurably affect KRAS interaction with RAF1 in cells, consistent with our structural data indicating that KRAS position 151 does not directly contact the RAF1 CRD (Fig. 7C). For position 153, we generated constructs in which the native amino acid of each isoform was replaced with the native amino acid of the other isoform (KRAS4a-E153D and KRAS4b-D153E). We then conducted BRET saturation curve experiments to compare each mutant to its respective WT isoform (fig. S12, B and C). Substitution at position 153 resulted in a significantly increased BRET_max_ but no significant change in BRET_50_ compared to the respective WT isoform (KRAS4a-E153D versus WT KRAS4a and KRAS4b-D153E versus WT KRAS4b) (table S2). This suggests that the substitutions lead to higher populations of KRAS-RAF1 complexes on the membrane without an increase in complex affinity. We note that direct comparisons between the results for each mutant are not appropriate because of the presence of isoform-specific HVRs in each construct, as well as additional isoform-specific amino acids on helix α5 of the G-domain.

## DISCUSSION

The growing interest in KRAS4a, following years of focus on KRAS4b, is attributed to the increasing recognition that this isoform, which was previously considered minor, plays a notable role in different tissues and developmental stages. Previous studies and this work reveal that KRAS4a transcriptional activity is substantial in many tissues (*9*), which mirrors proteomic data from numerous human and mouse cell lines that detected the KRAS4a protein in amounts comprising an average of 22.5% of total KRAS abundance (*31*). Our analysis of the TCGA dataset suggests that KRAS4a transcriptional activity is considerable in tissues such as the bile ducts, liver, and stomach, and is particularly relevant in certain tissues such as the colon and rectum, where its transcription approaches KRAS4b levels (Fig. 1C). Furthermore, it was demonstrated that the regulation of RAS isoforms extends beyond the spatial disparities observed in different tissue types and encompasses temporal regulation throughout an organism’s entire lifespan (*32*). Moreover, studies have indicated that among the four canonical RAS isoforms, KRAS4a expression exhibits the most dynamic regulation (*7*). Our analysis supports this finding by indicating that KRAS4a serves as the transcriptional target for dynamic regulation across various types of TCGA tumors (Fig. 1D). This observation suggests that tumors in different contexts tend to selectively manipulate the expression levels of KRAS4a to gain a survival advantage. A recent study investigating the RAS oncogene family’s origin and evolution suggested that KRAS4b is the primordial member of the RAS proto-oncogene family (*14*). Its duplication gave rise to HRAS in the common ancestor of vertebrates, which was then duplicated to form NRAS. KRAS4a was created by duplicating and inserting the fourth exon of NRAS into KRAS. The conservation of the KRAS4a and KRAS4b isoforms over 400 million years of evolution can be attributed to their functional significance and the influence of selective pressures (*14*). These evolutionary constraints encompass differential expression, distinct cellular and tissue-specific functions, and regulatory interactions.

In this study, we performed biochemical and biophysical characterizations of KRAS4a and KRAS4b to identify their similarities and differences and their impact on signaling. The melting temperature of KRAS4a is approximately 5°C lower than that of KRAS4b in both inactive and active states despite the very high sequence identity of the G-domain in the two isoforms. Our structure-function studies suggest that the isoforms’ sequence difference at position 151 mainly contributes to the observed difference in thermal stability by modulating the dynamic properties of helix α5. Amino acid 151 marks the beginning of the fourth exon (4a and 4b) and serves as the first point of divergence between the two KRAS isoforms—an arginine in KRAS4a and a glycine in KRAS4b. It is also the N-terminal cap of helix α5, meaning that it is the residue immediately preceding the first residue that has α-helical (φ,ψ) angles. In general, glycine is known to be more favorable as an N-cap than arginine due to the greater flexibility afforded by its reduced steric hindrance, which allows the subsequent residue to adopt an α-helical conformation more easily (*33*). In addition, the side chain of R151 in KRAS4a can interact with nearby residues Q150 and D154, thus further restricting the flexibility of the protein chain at the N terminus of helix α5. In KRAS4a and KRAS4b, the second sequence distinction occurs at position 153, where KRAS4a contains a glutamate, while KRAS4b has an aspartate residue. This residue plays a crucial role in the binding of the RAF1 CRD. The longer side chain of the glutamate in KRAS4a causes a moderate displacement of the RAF1 CRD by approximately 1.5 to 3 Å compared to its position when bound to KRAS4b (*27*). Notably, the native HRAS isoform also features a glutamate at position 153, and the HRAS:RAF1 CRD structure (*28*) displays a similar displacement of the CRD comparable to what is observed with KRAS4a. Therefore, residue 153 seems to finely adjust the positioning of the RAF1 CRD when interacting with RAS. Our study elucidates the biophysical and structural distinctions between the two KRAS isoforms, which can be directly linked to sequence variations in the G-domain. Notably, we found that the amino acid at position 151 has a pronounced impact on thermal stability, while position 153 governs the relative positioning of the bound RAF1 CRD.

To complement our structural analysis of KRAS4a and KRAS4b in the crystalline state, we analyzed their conformations in solution using NMR spectroscopy. The ^1^H-^15^N heteronuclear single-quantum coherence (HSQC) assignments of KRAS4a-GDP revealed residues with multiple amide peaks, indicating that the protein adopts multiple slowly exchanging conformations in solution. These multiple amide peaks originate from the conformationally exchanging residues in the switch regions and helices α3 and α5. No residues with multiple amide peaks were observed in KRAS4b-GDP despite its high sequence identity with KRAS4a. An analysis of KRAS4a in the active, GMPPNP-bound state yielded additional structural snapshots exhibiting subtle structural differences from the crystal structure. The RDC-refined structures display an expanded conformation compared to the crystal structures, exhibiting snapshots with conformational variability in key regions such as α1 (linked to switch I), the β2-β3 hairpin loop, switch II, α4, and the β6-α5 segment. Refining structures with solution data contributes additional structural snapshots that result in a more complete description of the conformational space that the protein can access. This in turn provides more accurate starting points available to computational biologists and medicinal chemists to efficiently design candidate drug compounds.

Germline mutations in the *KRAS* gene have been observed at various locations, including in exon 4 (V152G, D153V, and F156L) (*34*). These mutations are associated with developmental disorders known as RASopathies, which include Noonan syndrome (NS) and cardiofaciocutaneous syndrome. Residues V152 and F156 in the fourth exon are conserved in the two KRAS isoforms, whereas residue 153 is glutamate in KRAS4a and aspartate in KRAS4b. Biochemical characterization revealed that these mutants, like many oncogenic mutations, result in aberrant signaling via elevated levels of active, GTP-bound KRAS (*35*). The V152G mutation likely destabilizes the tertiary structure of KRAS as the presence of glycine inside the α5 helix disrupts its helical structure. The D153V mutation alters the electrostatic properties of the α5 helix by introducing a hydrophobic residue on its surface. The F156L mutation has been shown to increase intrinsic and SOS1-mediated nucleotide exchange and resistance to GAPs (*35*), likely by creating a cavity within the hydrophobic core, resulting in the loss of contact with surrounding residues. Structural destabilization caused by these mutations is likely to reduce the overall affinity for nucleotide binding. Because the cellular concentration of GTP is higher than that of GDP, these mutations lead to an increased population of active, GTP-bound RAS. The presence of RASopathy mutations in the α5 helix of the G-domain originating from exon 4 further emphasizes the substantial role of this region in RAS biology and disease development.

KRAS4a and KRAS4b rely on their distinct HVR sequences to be correctly recognized and trafficked to the PM via separate mechanisms. BRET saturation experiments are performed in live cells, yielding a quantitative readout of protein-protein interactions in an environment incorporating many features of a membrane-localized protein’s native physiological setting, such as lipid dynamics, protein posttranslational modifications, and the presence of multiple competing interaction partners in the surroundings. The higher BRET_max_ of KRAS4a observed in our assay could be interpreted as a greater number of total interacting pairs due to the differential localization of KRAS4a and KRAS4b to the subcompartments of the PM with varying effector concentrations. Alternatively, differences in orientation relative to the membrane may account for this increase, as changes in positioning between donor and acceptor tags affect the BRET signal (*36*, *37*). When the HVRs were swapped between KRAS constructs, the difference in BRET_max_ was reversed in p110α interactions and, to a lesser extent, in RAF1 interactions (Fig. 8, E and F). This finding emphasizes the HVR’s influence on RAS’s surrounding microenvironment and interaction with downstream effectors when observed in a cellular context. Future studies need to explore in greater detail the role of the isoforms’ HVR in regulating RAS-effector interactions. Our BRET data reveal that position 153 on helix α5, in addition to the HVR, influences interaction with RAF1 within cells. Substituting the residue at position 153 in each isoform with the one native to the other isoform significantly enhances BRET_max_ without altering BRET_50_ values compared to the respective WT isoform. The interpretation of mutant data at position 153 is complex due to the presence of isoform-specific HVRs in each construct, along with other amino acid variations on helix α5 of the G-domain. The observed changes in BRET_max_ may also result from various other factors, such as altered protein trafficking and membrane localization, distinct electrostatic interactions with membrane lipids affecting orientation relative to the membrane, or reduced affinity for competing effectors, thereby increasing the availability of KRAS to bind a specific effector.

The generation of alternative splice variants, a characteristic of most multi-exon genes, may account for the apparent paradox that many complex species such as mammals often have a smaller number of protein-encoding genes than life forms considered less complex, yet display greater phenotypic complexity (*38*, *39*). The relative abundance of *KRAS* splice variants is governed by the efficiency of alternative splicing, which remains poorly understood in terms of its regulatory mechanisms. A recent study demonstrated that the DCAF15/RBM39 pathway regulates the splicing of *KRAS4a*. Furthermore, the deletion of *KRAS4a* or pharmacological inhibition of RBM39 using the splicing inhibitor indisulam has been shown to suppress cancer stem cells (*40*). Amino acid sequence analysis of exons 4a and 4b in various organisms revealed considerable conservation of residues within the G-domain, specifically spanning positions 151 to 166, for both KRAS4a and KRAS4b, throughout the course of evolution (fig. S11). However, notable sequence divergence was observed in 4b exon among organisms, primarily in the CaaX motif, residues 180 to 184 involved in interactions with PDEδ, and the PBR. Similarly, 4a exon in different organisms also exhibits divergence in the CaaX motif, the KIKK motif present in PBR1, and residues 169 and 171 to 173 located downstream of PBR2. The conservation of most residues in the fourth exon of both KRAS4a and KRAS4b underscores their importance in the distinct functions of these two isoforms. Meanwhile, the minor variations in residues within the HVR suggest varying levels of regulation of membrane trafficking or anchoring among different organisms during evolution.

Considering the differential modification and trafficking of KRAS isoforms, studies have been conducted to investigate the unique interactomes of the two splice variants of KRAS. Zhang *et al.* (*10*) examined the nucleotide-dependent interactomes of KRAS4a and KRAS4b, identifying several previously unknown interacting proteins, some of which interacted with both isoforms, while others showed specificity toward one isoform. Notably, KRAS4b displayed a specific interaction with v-ATPase (adenosine triphosphatase) a2, which plays a key role in regulating KRAS-induced micropinocytosis on the cytosolic face of lysosomes, aligning with the localization of KRAS4b. This suggests that the differential intracellular localizations of KRAS4a and KRAS4b contribute to the distinct protein-protein interactions observed between them. Further support for this notion was found in the association of KRAS4a with hexokinase 1 on the outer mitochondrial membrane, a relationship absent in the KRAS4b interactome (*3*). In addition, the selectivity of Sin1 toward KRAS4a likely relies on the HVR. The fusion of the G-domain of KRAS4b with the KRAS4a HVR facilitated interaction with Sin1, whereas the fusion of the KRAS4b HVR with the KRAS4a G-domain significantly impaired the interaction (*4*). Thus, the distinct protein-protein interactions of KRAS4a and KRAS4b can be attributed to their differential intracellular localizations, as supported by specific interactions with v-ATPase a2, hexokinase 1, and Sin1. A recent study showed that, unlike KRAS4b, the HVR of KRAS4a undergoes lysine fatty acylation, a posttranslational protein modification that has received less exploration (*11*). Sirtuin 2 (SIRT2), a mammalian nicotinamide adenine dinucleotide–dependent lysine deacylase, removes fatty acylation from KRAS4a. Depletion of SIRT2 results in increased lysine fatty acylation, leading to a decrease in the transforming activity of KRAS4a (*11*).

The existence of hotspot oncogenic mutations at positions 12, 13, and 61 in both KRAS4a and KRAS4b presents a major obstacle for targeted therapies because of differences in their structures and functions. With the presence of two splice variants, the complexity of resistance mechanisms also increases, emphasizing the need to identify strategies to overcome or prevent resistance in both KRAS4a and KRAS4b. This study provides a comprehensive biochemical and structural analysis of KRAS4a and compares it with KRAS4b, revealing notable differences in their structural properties and thermal stability. Specifically, the first amino acid of the fourth exon, R151 in KRAS4a and G151 in KRAS4b, plays a crucial role in the dissimilarities observed in the G-domain. In addition, our BRET experiments yielded additional evidence highlighting the contrasting activation patterns of downstream signaling pathways between the two KRAS isoforms due to their divergent HVRs. Future studies should focus on delineating the distinct signaling properties of KRAS4a and KRAS4b to develop novel therapeutic approaches that effectively target both splice variants.

## MATERIALS AND METHODS

### Preparation of protein expression constructs

Expression clones KRAS4a(1–177) and R151G, R151A, E153D, Y166H, Q165K, R167K, L168E point mutants, KRAS4a(1–169) and R151G variant, KRAS4b(1–169)G151R, and PIK3CG(144–1102)-His6 V223K were generated by ATUM via synthesis of Gateway entry clones optimized for expression in *Escherichia coli* (RAS) or insect cells (PIK3CG). Entry clones for RAS were then subcloned into pDest-566 (Addgene, no. 11517) to produce final expression clones containing RAS with an N-terminal His6-MBP (maltose-binding protein) fusion. The entry clone for PIK3CG was subcloned into pDest-602, a modified version of pFastBac-1 (Thermo Fisher Scientific) containing an N-terminal MBP fusion. The PIK3CG expression clone was converted into a baculovirus bacmid vector using the Bac-to-Bac system (Thermo Fisher Scientific) using the manufacturer’s instructions. The expression clone and subsequent bacmid DNA for PIK3Cα, R724-M79-623, were generated in a similar manner with exceptions. Specifically, an upstream tobacco etch virus (TEV) protease site (ENLYFQ/G) was incorporated by PCR (template was R724-T02, encoding Hs.PIK3Cα, provided by the Amzel laboratory) and Gateway multisite (*41*) was used to combine expression cassettes for His6-MBP-tev-PIK3Cα and p85 into Dest-623 (Addgene, no. 161878). Expression clones for the production of PDEδ (*30*), SOS1 catalytic domain (564 to 1048) (*42*), RAF1 (52 to 131) (*43*), NF1GAPiso2 (1198 to 1530) (*42*), RAF1 (52 to 131) (*43*), NF1GAPiso2 (1198 to 1530) (*42*), Gly-Hs.KRAS4b (1 to 169) (*42*), and RAF1 (52 to 188) (Addgene, no. 159697) have been described previously.

### Protein expression

His6-MBP-tev-Gly-Hs.KRAS4a(1–177) and mutants, His6-MBP-tev-Gly-Hs.KRAS4a(1–169), His6-MBP-tev-Gly-Hs.KRAS4a(1–169)R151G, His6-MBP-tev-Gly-Hs.KRAS4b(1–169), His6-MBP-tev-Gly-Hs.KRAS4b(1–169)G151R, His6-MBP-tev-PDEδ, His6-tev-SOScat, and His6-MBP-tev-NF1GAP(1198–1530) were expressed as previously published using Dynamite media with induction at 16°C (*44*). His6-MBP-tev-RAF1(52–188) was expressed as described by Travers *et al.* (*45*) with modifications. Specifically, *Vibrio natriegens* was the expression host (VMAX X2, Telesis Bio, San Diego, CA), incubation was done at 30°C for all culture steps, and the overnight seed medium was ZYM 20050 plus 1.5% (w/v) Instant Ocean Sea Salt (Instant Ocean, Spectrum Brands, Blacksburg, VA), minus lactose, shaken at 250 rpm. After 12.5 hours, 50 ml of seed culture was used to inoculate 2 liters of the modified ZYM 20050 medium, agitation rate of 481 rpm, airflow of 2.5 liters per minute (LPM), induction at an optical density at 600 nm (OD_600_) of 6.0 with 1.0 mM isopropyl-β-d-thiogalactopyranoside (IPTG), and induction time of 8.0 hours. His6-MBP-tev-Hs.RAF1(52–131) was expressed as described by Taylor *et al.* (*44*) with the autoinduction protocol with ZYM medium.

For the ^15^N incorporation, His6-MBP-tev-Gly-Hs.KRAS4a(1–169), His6-MBP-tev-Gly-Hs.KRAS4b(1–169), and His6-MBP-tev-Gly-Hs.KRAS4a(1–169)R151G, were expressed as described by Agamasu *et al.* (*46*) with modifications. Specifically, the overnight seeds for the KRAS4a constructs were grown in 300 ml of MDAG medium in 2-liter baffled flasks at 37°C, 250 rpm, and the collected pellets were resuspended in 300 m of T-20052 medium and used to inoculate 15 liters of T-20052 medium in separate 20-liter BioFlow IV bioreactors (Eppendorf/New Brunswick Scientific, Edison, NJ) at 37°C, airflow of 15 LPM, and agitation rate of 350 rpm. For the KRAS4b construct, an overnight seed was grown in 200 ml of MDAG medium in a 2-liter baffled flask, at 37°C, 250 rpm, and the collected pellet was resuspended in 200 ml of T-20052 medium and used to inoculate 10 liters of T-20052 medium in a BioFlow 3000 bioreactor (Eppendorf/New Brunswick Scientific, Edison, NJ) at 37°C, with airflow of 10 LPM and agitation rate of 400 rpm.

For the ^13^C/^15^N incorporation, His6-MBP-tev-Gly-Hs.KRAS4b(1–169) was expressed as described by Travers *et al.* (*45*) with modifications. Specifically, the overnight *E. coli* seed was grown in 300 ml of MDAG; the collected pellet was resuspended in 100 ml of Mod M9 medium (without additional zinc chloride) and used to inoculate 15 liters of Mod M9 medium in a BioFlow IV bioreactor at 37°C, airflow of 15 LPM, agitation rate of 350 rpm, until an OD_600_ of 0.5, and the temperature shifted to 16°C for overnight induction.

For the ^13^C/^15^N incorporation, His6-MBP-tev-Gly-Hs.KRAS4a(1–169) was expressed as described by Travers *et al.* (*45*) with modifications. Specifically, *V. natriegens* was the expression host, and seed culture was in 300 ml of ZYM medium with 1.5% (w/v) Instant Ocean, no lactose or NaCl, and grown overnight at 30°C in a 2-liter baffled flask. The collected pellet was resuspended in 300 ml of ModM9 with NaCl (15 g/liter) and no lactose, used to inoculate 15 liters of the same medium in a 20-liter BioFlow IV bioreactor, and grown at 30°C, with airflow of 18.75 LPM and agitation rate of 350 rpm, to an OD_600_ of 0.5 (~2 hours), shifted to 25°C, induced with 1.0 mM IPTG, and harvested after 21 hours.

### Protein purification and nucleotide exchange

All proteins except PIK3CA(1–1068) W1057A/I1058A/F1059A + PIK3R1(322–600) and PIK3CG(144–1102)-H6 V223K were purified essentially as described (*47*), omitting MgCl_2_ for all non-RAS proteins. PIK3CA(1–1068) W1057A/I1058A/F1059A + PIK3R1(322–600) and PIK3CG(144–1102)-H6 V223K was purified as described previously with modifications (*47*). Specifically, for PIK3CG(144–1102)-H6 V223K, the buffer for lysis and immobilized metal affinity chromatography (IMAC) steps was 20 mM tris-HCl ( pH 8.0), 300 mM potassium glutamate, and 1.0 mM tris(2-carboxyethyl)phosphine (TCEP). The clarified lysate was adjusted to 25 mM imidazole before loading on an IMAC column equilibrated in the lysis buffer (buffer adjusted to 25 mM imidazole). An anion-exchange step was included after the second IMAC step. The protein pool from the second IMAC step was dialyzed to 20 mM tris-HCl (pH 8.0), 75 mM potassium glutamate, and 1.0 mM TCEP (buffer A), and loaded on a Q Sepharose High-Performance column (Cytiva) equilibrated in buffer A. The protein was eluted with a 10-column volume gradient from 0 to 50% buffer B (buffer A + 500 mM NaCl). The buffer for the size exclusion chromatography step was 20 mM tris-HCl (pH 8.0), 150 mM NaCl, and 1.0 mM TCEP. PIK3CA(1–1068) W1057A/I1058A/F1059A + PIK3R1(322–600) followed a similar protocol except that the lysis buffer and IMAC steps used 20 mM tris-HCl (pH 8.0), 150 mM NaCl, and 1.0 mM TCEP, and the protein pool from the second IMAC was dialyzed into 20 mM tris-HCl (pH 8.5) and 1.0 mM TCEP (buffer A2); this buffer was used for the Q Sepharose High-Performance column (Cytiva). GDP bound to RAS proteins was exchanged for GMPPNP as described previously (*43*), with the modification that the final incubation with MgCl_2_ and GMPPNP was performed overnight at 4°C instead of for 1 hour at room temperature.

### Crystallization and structure determination

All KRAS crystallographic experiments in the present work used constructs containing residues 1 to 177 except for KRAS4a-R151G, which contained residues 1 to 169. Crystallization trials were carried out via vapor diffusion in sitting drops. The buffer for all proteins and protein complexes consisted of 25 mM Hepes (pH 7.4), 150 mM NaCl, 5 mM MgCl_2_, and 1 mM TCEP, and crystallization drops contained equal volumes of protein solution and well solution. WT KRAS4a-GDP was crystallized with a well solution containing 0.2 M ammonium iodide and 2.2 M ammonium sulfate. WT KRAS4a-GMPPNP was crystallized in a well solution of 0.2 M NaBr and 20% (w/v) polyethylene glycol (PEG) 3350. The KRAS4a-R151G-GDP mutant was crystallized in a well solution of 100 mM Hepes (pH 7.4), 2 M ammonium sulfate, and 0.1 M MgCl_2_. Initial crystals of WT KRAS4a-GMPPNP bound to the RAF1 RBD (residues 52 to 131) were obtained with a well solution of 0.2 M sodium potassium phosphate (pH 7.4), 22% (w/v) PEG 3350, and 0.1 M bis-tris propane (pH 7.5); these poor crystals were crushed and used as seeds in random microseed matrix screening (*48*), which yielded a diffraction-quality crystal in a well solution of 0.15 M dl-malic acid (pH 7.0) and 20% (w/v) PEG 3350. Initial crystalline needles of the WT KRAS4a-GMPPNP:RAF1 RBD-CRD (residues 52 to 188) complex were obtained in a well solution of 0.1 M ammonium sulfate, 0.3 M sodium formate, 5% (w/v) PEG 8000, 3% (w/v) poly-γ-glutamic acid, and 0.1 M sodium cacodylate (pH 6.5); these initial crystals were crushed and used as seeds in random microseed matrix screening, which yielded a diffraction-quality crystal in a well solution of 0.1 M sodium acetate (pH 4.6) and 2 M sodium formate.

All diffraction data were collected at Northeastern Collaborative Access Team (NE-CAT) beamlines 24-ID-C/E of the Advanced Photon Source at Argonne National Laboratory (Lemont, IL, USA) using x-rays of wavelength 0.9791 Å and either a Dectris PILATUS 6M-F or a Dectris EIGER 16M detector. Data reduction and scaling were performed using XDS (*49*). Phasing of all structures was accomplished by molecular replacement in Phaser (*50*) using chain A from PDB 6VC8 as the search model for KRAS4a and residues 52 to 131 or 52 to 188 of chain B of PDB 6XI7 as the search model for RAF1 RBD or RBD-CRD, as appropriate. Molecular replacement solutions were rebuilt using phenix.autobuild, and the rebuilt solutions were iteratively updated by manual model building in Coot (*51*) and refinement with phenix.refine (*52*). Although all constructs except KRAS4a-R151G ended at residue 177, interpretable electron density typically ended around residue 170. Comprehensive data collection and refinement statistics are presented in Table 1. All structural figures were prepared in PyMOL version 2.5 (Schrödinger Inc.). Electrostatic surface calculations were performed using the Adaptive Poisson-Boltzmann Solver (*53*) plugin for PyMOL.

### NMR spectroscopy

All NMR experiments were performed on KRAS constructs containing residues 1 to 169. KRAS4a and KRAS4b NMR samples contained 0.25 to 1.2 mM uniformly [^15^N]- or [^13^C/^15^N]-labeled protein bound to either GDP or GMPPNP in a buffer consisting of 20 mM Hepes (pH 7.4), 150 mM NaCl, 1 mM TCEP, and 5% D_2_O in susceptibility-matched 5-mm Shigemi microtubes. Anisotropic samples additionally contained either Pf1 phage particles (20 mg/ml; Asla Biotech, Riga, Latvia) or 5% (w/v) of *q* = 3.0 1,2-dimyristoyl-sn-glycero-3-phosphocholine (DMPC)/1,2-dihexanoyl-sn-glycero-3-phosphocholine (DHPC) bicelles (Avanti Polar Lipids, Alabaster, AL, USA). ^31^P NMR spectra were acquired at 5°C using a Bruker 500 MHz NMR spectrometer (202-MHz ^31^P frequency) equipped with a 5-mm Prodigy broadband cryogenic probe using 70° flip angle pulses, 1200 scans, an interscan delay of 7 s, an acquisition time of 84 ms, and a WALTZ-16 proton decoupling sequence. The rest of the NMR spectra were acquired on a Bruker 700-MHz NMR spectrometer at 25°C, except for the RDC spectra in bicelles, which were measured at 34°C.

Resonance assignments of KRAS4a in both the GDP- and GMPPNP-bound states were obtained from analysis of a standard set of 2D and 3D NMR spectra: 2D ^1^H-^15^N HSQC, 3D HNCACB, 3D CBCA(CO)NH, and 3D C(CO)NH. All 3D experiments were recorded using nonuniform sampling at a sampling rate of 40%, and the spectra were reconstructed using the SMILE (*54*) and NMRPipe (*55*) software packages. Assignments were performed using NMRFAM-Sparky (*56*) and deposited in the BMRB (entries 52001 and 52002). Virtually all non-proline amide signals were assigned in the GDP state (164 of 165 amides, 99.4% completeness; figs. S4 and S5). In the GMPPNP-bound state, we assigned 124 amides out of 165 non-proline residues (fig. S6). As frequently observed in the GMPPNP-bound state of KRAS, the amides in the two dynamic switch regions (residues 29 to 41 and 57 to 73) are undetectable because of complete dynamic peak broadening in all NMR spectra (intermediate exchange). When these dynamic switch regions are excluded, the assignment completeness percentage is 91.9%. ^1^D_NH_ and ${}^{1}D_{C^{\alpha }H^{\alpha }}$ RDCs were measured using a 3D-HNCO variant of the ARTSY (*57*) experiment and a 3D HCA(CO)N experiment with antiphase ^1^H-coupling in the ^13^C^α^ dimension, respectively. All RDC experiments were acquired with sufficient S/N to ensure low experimental errors illustrated by the error bars in Fig. 6 and fig. S8 (some become smaller than the dot size for experimental RDCs with large ranges of magnitude). We used Xplor-NIH (*58*) to refine KRAS4a x-ray structures in torsion angle space against RDC and Talos-N (*59*) dihedral angle restraints, allowing up to 3-Å departures from the initial (x-ray) heavy atom (CA, C, N) positions in a non-crystallographic symmetry (NCS)-type potential and an additional C^α^-C^α^ distance restraint potential. Since no RDCs could be measured for the two dynamic switch regions (residues 29 to 41 and 57 to 73), no experimental restraints acted on these flexible regions during structural refinement, so they remained in their starting crystal structure conformation. The 20 lowest-energy structures refined with RDCs have an RMSD of 1.1 Å, while the RMSD between the average of these structures and the starting x-ray structural model is 2.0 Å. All 3D structural representations were made in PyMOL (Schrödinger Inc.).

### DSF measurements

The thermal melting temperature (*T*_m_) of various KRAS4a and KRAS4b mutants (residues 1 to 169 or 1 to 177) was measured using an Applied Biosystems QuantStudio 3 real-time PCR instrument. The reaction replicates for the assay were assembled in a MicroAmp Optical 96-well reaction plate; in each replicate, the protein concentration was 1 mg/ml in a buffer of 20 mM Hepes (pH 7.4), 150 mM NaCl, 5 mM MgCl_2_, and 1 mM TCEP. Each reaction replicate was made by mixing 18 μl of protein stock and 2 μl of 10× Sypro Orange dye (Thermo Fisher Scientific, no. 4462263) in each well. Plates were sealed with optically clear adhesive film (Thermo Fisher Scientific, no. 4311971) and centrifuged at 720*g* for 2 min before insertion into the thermal cycler. After incubating the plate at 25°C for 2 min, the fluorescence was measured continuously while ramping the temperature from 25° to 99°C at a rate of 0.05°C/s. The acquired data were analyzed using the Applied Biosystems Protein Thermal Shift Software to extract the *T*_m_ value. The result for each protein is reported as the mean measured *T*_m_ ± SD (*n* = 3).

### ITC measurements

For measuring binding affinities using ITC, protein samples for all experiments except for KRAS4a/b binding to PI3Kα were prepared by extensive dialysis in a buffer (filtered and degassed) containing 20 mM Hepes (pH 7.3), 150 mM NaCl, 5 mM MgCl_2_, and 1 mM TCEP. For the experiments with PI3Kα, the proteins were dialyzed overnight against 20 mM tris (pH 7.8), 150 mM NaCl, 5 mM MgCl_2_, and 1 mM TCEP. For the binding of the KRAS4a(167–186)HVR-FMe peptide (AnaSpec Peptides Inc., Freemont, CA) to PDEδ, the peptide was dissolved in an aliquot of the buffer in which PDEδ was dialyzed. Before being loaded into the calorimeter, all protein and peptide samples were centrifuged at 14,000*g* for 5 min. For the ITC measurements, 60 μM KRAS and 600 μM of either RAF1-RBD, PI3Kα, or PI3Kγ were placed in the cell and syringe, respectively. For the binding experiment with the KRAS4a-HVR peptide and PDEδ, the syringe contained 1 mM KRAS4a(167–186)HVR-FMe peptide, while the cell contained 200 μM PDEδ. The titrations were performed at 25°C using a MicroCal PEAQ-ITC instrument (Malvern), with an initial injection of 0.4 μl and 18 subsequent injections of 2.2 μl at intervals of 150 s. The data were fit to a single binding site model using the nonlinear least squares algorithm in the MicroCal PEAQ-ITC analysis software (Malvern).

### Intrinsic and GAP-mediated GTP hydrolysis assay

KRAS4a and KRAS4b proteins (residues 1 to 169) were exchanged from the GDP- to GTP-bound state, as previously described (*60*), and the efficiency of the exchange was measured by high-performance liquid chromatography (HPLC) (*46*). KRAS proteins (3 μM final concentration) were diluted into 50 mM tris-HCl (pH 7.5), 1 mM dithiothreitol, 150 mM NaCl, and 2 mM MgCl_2_ containing 4.5 μM phosphate-binding proteins labeled with 7-Diethylamino-3-[N-(2-maleimidoethyl)carbamoyl]coumarin (MDCC) (Phosphate Sensor, Fisher Scientific) for intrinsic GTPase activity or containing 10, 1, or 0.1 nM NF1-GAP333iso2 for GAP-mediated GTPase activity in a final volume of 40 μl per well. All reactions were performed in quadruplicate, in a black, clear-bottom 384-well plate and read in a BioTek Neo2 plate reader at ambient temperature (~25°C) using excitation and emission wavelengths of 430 and 450 nm.

### Intrinsic and SOS-mediated nucleotide exchange assay

WT KRAS4a and KRAS4b proteins (residues 1 to 169) were prepared in 20 mM Hepes, 150 mM NaCl, 2 mM MgCl_2_, and 1 mM TCEP (assay buffer) and were exchanged from GDP to N-Methylanthraniloyl (MANT)-GDP using a protocol described previously (*60*). Nucleotide exchange efficiency was determined to be >90% via a previously published ion-pairing reversed-phase HPLC method (*46*). MANT-GDP–exchanged protein concentrations were determined using a NanoDrop spectrophotometer. KRAS4a or KRAS4b (1.5 mM) was diluted in the assay buffer with variable SOS1 catalytic domain (564 to 1048) concentrations (0, 0.125, 0.25, 0.5, and 1 mM) and 1.5 mM GDP in a final volume of 200 ml. Each reaction was prepared in duplicate in a black, clear-bottomed, 96-well plate. The plates were read in a BioTek Neo2 plate reader at room temperature (~25°C) with an excitation wavelength of 355 nm and an excitation wavelength of 448 nm.

### Cell culture and DNA constructs

Human embryonic kidney (HEK) 293T cells (American Type Culture Collection, CRL-3216) were cultured in complete phenol red–free Dulbecco’s modified Eagle’s medium (DMEM; Life Technologies, 31053-028, 4.5 g/liter glucose, 3.7 g/liter sodium bicarbonate), 2 mM l-glutamine (Life Technologies, 25030081), and 10% fetal bovine serum (FBS; Hyclone, SH30071.03) at 5% CO_2_ and 37°C. NanoLuc and mVenus fusion constructs for mammalian expression were generated by Multisite Gateway recombinational cloning. Most Gateway entry clones used can be obtained from Addgene (table S3). All backbone vectors have a similar design to pDest-303 (Addgene, 159678) and pDest-305 (Addgene, 161895). pDest-304 is identical to pDest-305 but with hygromycin resistance replacing puromycin resistance. pDest-313 is the same vector as pDest-303 but uses a different Gateway site configuration for cloning. The third entry clones for the KRAS4b/4a chimeras were generated by synthesis (ATUM Inc.) to produce the appropriate chimeric KRAS4b and KRAS4a sequences. The third entry clones used for WT KRAS4a/b were generated by PCR from MGC cDNA (Horizon). Mutant KRAS4a entry clones were also generated by synthesis (ATUM Inc.) as well as mutagenesis using the QuikChange II site-directed mutagenesis kit (Agilent). Final DNA constructs were sequence-validated, and transfection-quality DNA was prepared using the HiSpeed Plasmid Maxiprep Kit (Qiagen Inc.).

### BRET assay

BRET saturation curves were achieved through cotransfection of constant concentrations of NanoLuc-tagged donor plasmid and increasing concentrations of mVenus-tagged acceptor plasmid constructs into HEK293T cells using Fugene 6 transfection reagent (Promega, E2691). The total mass of DNA was held constant by adding empty vector plasmid DNA as needed. Cells were seeded into 12-well plates (Falcon, 353043) at 1.25 × 10^5^ per well in 1 ml of complete 10% FBS–DMEM 18 hours prior. Transfections were incubated for 48 hours, after which the cells were resuspended using 0.25% trypsin-EDTA (Life Technologies, 25200-056) and complete media. Cells were then centrifuged, resuspended in 5% FBS–Dulbecco’s phosphate-buffered saline, and transferred to both a 384-well black (PerkinElmer, 6007270) and white (PerkinElmer, 6007290) Opti-Plate in triplicate at a density of 20,000 cells per 20 μl per well. mVenus (acceptor) signal from the black plate was read using Envision software with a yellow fluorescent protein (YFP) 500/20 (barcode 139) excitation and YFP 530/10 (barcode 221) emissions filter with a cyan fluorescent protein (CFP)/YFP mirror (barcode 428), and values from each point were averaged. The NanoLuc substrate furimazine (Promega, N1663) was added to the white plate for a final concentration of 10 μM and total volume of 40 μl. After exactly 10 min of room temperature incubation, the plate was read on Envision software with emissions filters YFP 530/10 (barcode 221) and 460 (barcode 502).

### BRET curve analysis

BRET ratios were determined by the BRET channel emissions (530 nm)/NanoLuc channel emissions (460 nm) for each point. milliBRET (mBRET) values were then generated with the following formula:

1000*(BRET ratio−average BRET ratio of donor only control)

The A/D value of each point was determined by dividing the average mVenus emissions by the NanoLuc emissions, including background measurements from the donor-only control. All points were normalized to the seventh point in the curve with the following equation:

(A/D value)−(background A/D)

(Control A/D)−(background A/D)

Curves were then plotted using GraphPad Prism software and fitted using the Hyperbola (X is concentration) nonlinear regression model. BRETmax and BRET50 values were extrapolated from the curve fits of three biological replicates, and a paired *t* test was performed to determine significance.

### Analysis of expression levels of KRAS isoforms in TCGA data

The data from TCGA, specifically the RNA-seq data at the transcript level of gene isoforms and the genome-wide mutation status data, were obtained and processed following the methodology described in a previous study (*61*). The normalized RNA-seq data, expressed as RSEM (RNA-seq by expectation-maximization) values at the transcript level, were used to generate percent stacked bar charts and boxplots specifically for KRAS4a and KRAS4b isoforms. Customized R scripts (https://r-project.org/) were used to perform these visualizations. The KRAS mutation status was retrieved for each tumor sample to categorize the samples within each tumor type. Various tests were conducted to assess the statistical significance of the findings, including nonparametric Kruskal-Wallis one-way analysis of variance (ANOVA) tests and parametric one-way ANOVA tests. Customized R scripts were used to execute these statistical analyses.

### Alignment of protein sequences of KRAS isoform from various selected species

Amino acid sequences of KRAS were obtained through three distinct methodologies. Initially, a total of 50 individual KRAS-like protein sequences were manually acquired from the National Center for Biotechnology Information (NCBI) protein database, focusing on model organisms with available sequences (referenced at: https://en.wikipedia.org/wiki/Model_organism, accessed on 6 July 2023). Subsequently, supplementary files from a preprint publication (https:/researchsquare.com/article/rs-2485219/v1) provided an additional 64 KRAS protein sequences from various species. Last, a sequence batch download procedure was conducted using NCBI databases and gene utilities to obtain KRAS orthologs. Following the combination of KRAS-like sequences obtained from these three approaches and removing redundant sequences, 92 sequences representing KRAS4a- and KRAS4b-like sequences were selected. These sequences originated from 38 distinct species across different GenBank divisions and divisions indicated by the NCBI taxonomy databases. Initial analyses involved iterative alignments and the creation of phylogenetic trees using both full protein sequences and specific sequence fragments spanning amino acids 151 to 189. These analyses encompassed all collected KRAS and KRAS-like sequences from selected species, including numerous model organisms. In cases where a species exhibited designated KRAS4a-like and KRAS4b-like sequences within our selected dataset, any additional isoforms with longer sequences or substantial divergence from the main KRAS4a and KRAS4b clusters were excluded. Specifically, sequence fragments spanning amino acids 151 to 189 were chosen for 30 KRAS4a-like isoforms within the KRAS4a cluster, while fragments spanning amino acids 151 to 188 were selected for 38 KRAS4b-like isoforms within the KRAS4b cluster. Individual alignments of KRAS4a or KRAS4b proteins for the selected model organisms were created using various alignment methods, including Clustal Omega, ClustalW, and Muscle. However, only results from Clustal Omega were presented, while the other methods were used for validation purposes. Sequence manipulation, editing, alignment, and phylogenetic tree construction were performed using customized R scripts (https://r-project.org/) and relevant R packages such as seqinc, msa, and ape, in addition to other data manipulation packages. The web version of Clustal Omega from EMBL-EBI (https://ebi.ac.uk/Tools/msa/clustalo/) was also used for exploring and validating the alignments. To enhance the readability of the species names, the original sequence names were replaced with descriptive annotations, including GenBank divisions and common names, which were retrieved from the NCBI taxonomy database for all species within our selected dataset, using customized R scripts.
