# Supplementary material for: Comparative analysis of KRAS4a and KRAS4b splice variants reveals distinctive structural and functional properties
Source: Sci Adv. 2024 Feb 14;10(7):eadj4137. doi: 10.1126/sciadv.adj4137 (PMC11636682; doi:10.1126/sciadv.adj4137)
Supplement: Supplementary file 1 — Figs. S1 to S12 Tables S1 to S3 [file sciadv.adj4137_sm.pdf]

Science Advances
AAAS

Supplementary Materials for

Comparative analysis of KRAS4a and KRAS4b splice variants reveals distinctive structural and functional properties

Matthew J. Whitley *et al.*

Corresponding author: Dhirendra K. Simanshu, dhirendra.simanshu@nih.gov;
Gabriel Cornilescu, gabriel.cornilescu@nih.gov

Sci. Adv. **10**, eadj4137 (2024)
DOI: 10.1126/sciadv.adj4137

This PDF file includes:

Figs. S1 to S12
Tables S1 to S3

Supplementary Figures

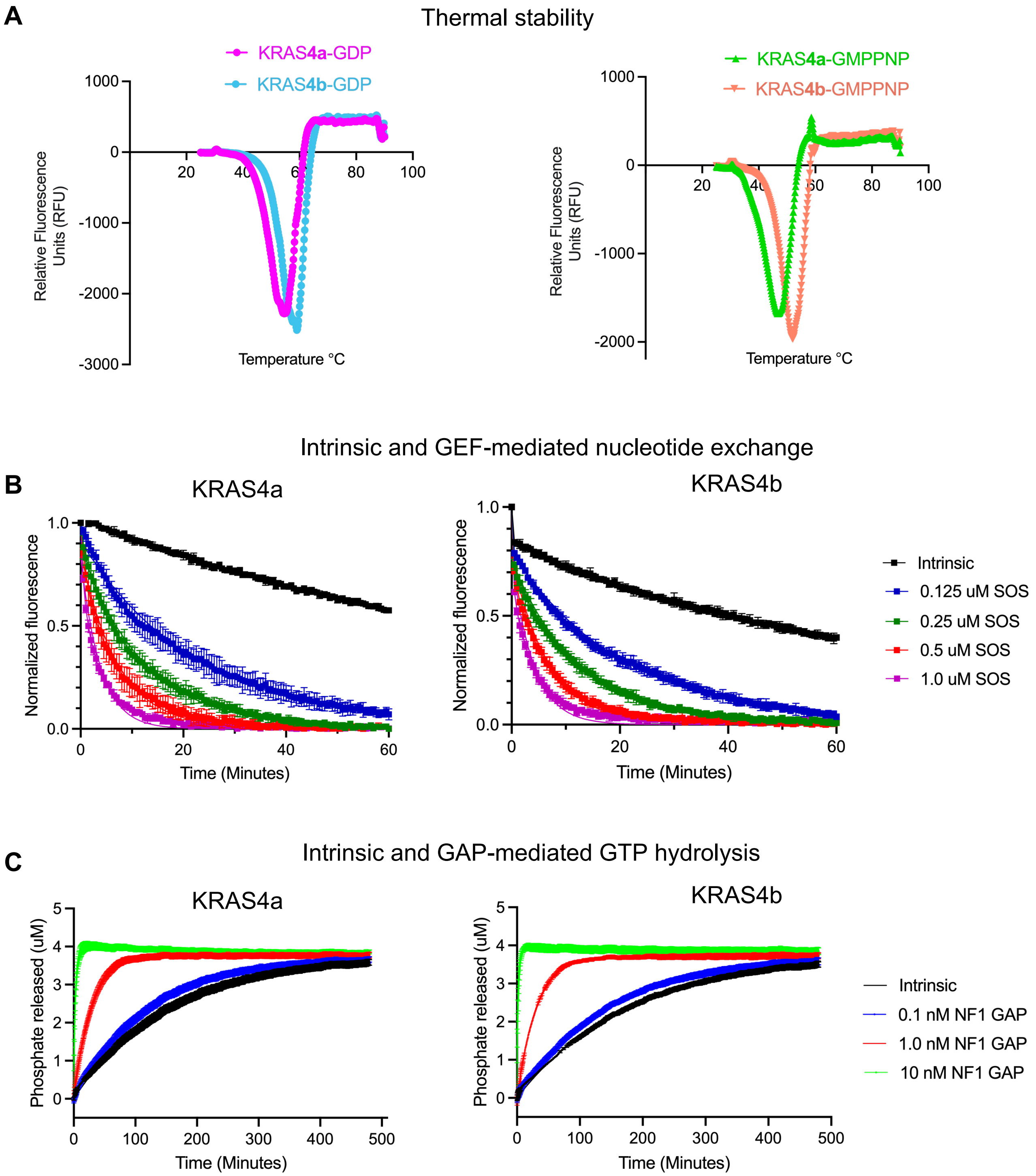

Fig. S1. Biophysical and biochemical comparison of KRAS4a and KRAS4b. A) The first-derivative plot of thermal melting data for KRAS4a and 4b in the inactive (GDP-bound) and active (GMPPNP-bound) states were obtained using differential scanning fluorimetry. The melting temperature (T_m) is the temperature at which the first-derivative curve reaches its minimum. B) Intrinsic and SOS1 (GEF)-stimulated GDP release by KRAS4a and KRAS4b. C) Intrinsic and NF1 (GAP)-mediated GTP hydrolysis by KRAS4a and KRAS4b.

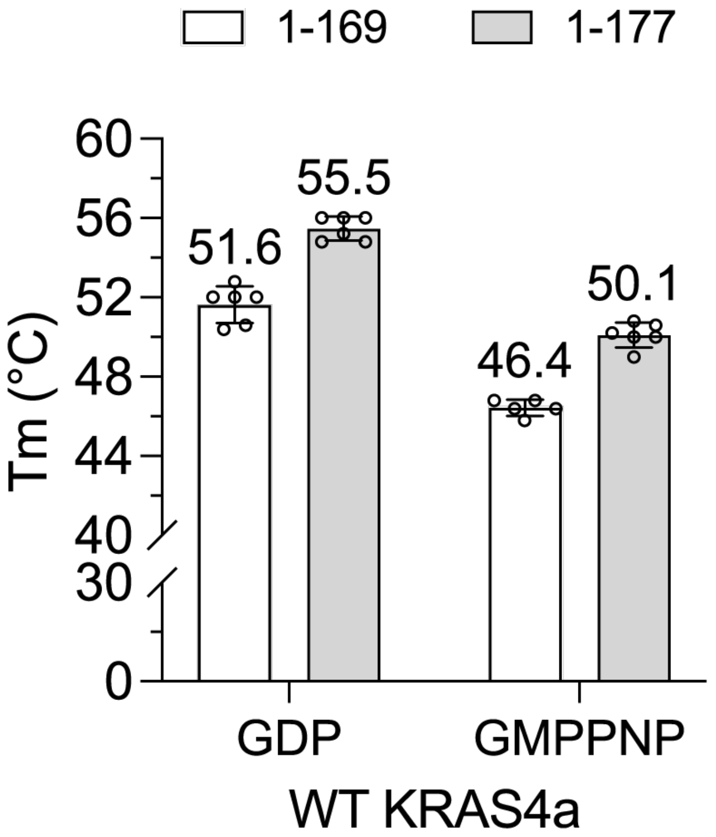

Fig. S2. Melting temperature (Tm) analysis as a function of bound nucleotide for the two different KRAS4a construct lengths (residues 1-169 and residues 1-177) used in this study. The open circles represent the individual data points, and the error bars represent the standard deviation calculated from 5 or 6 replicate measurements.

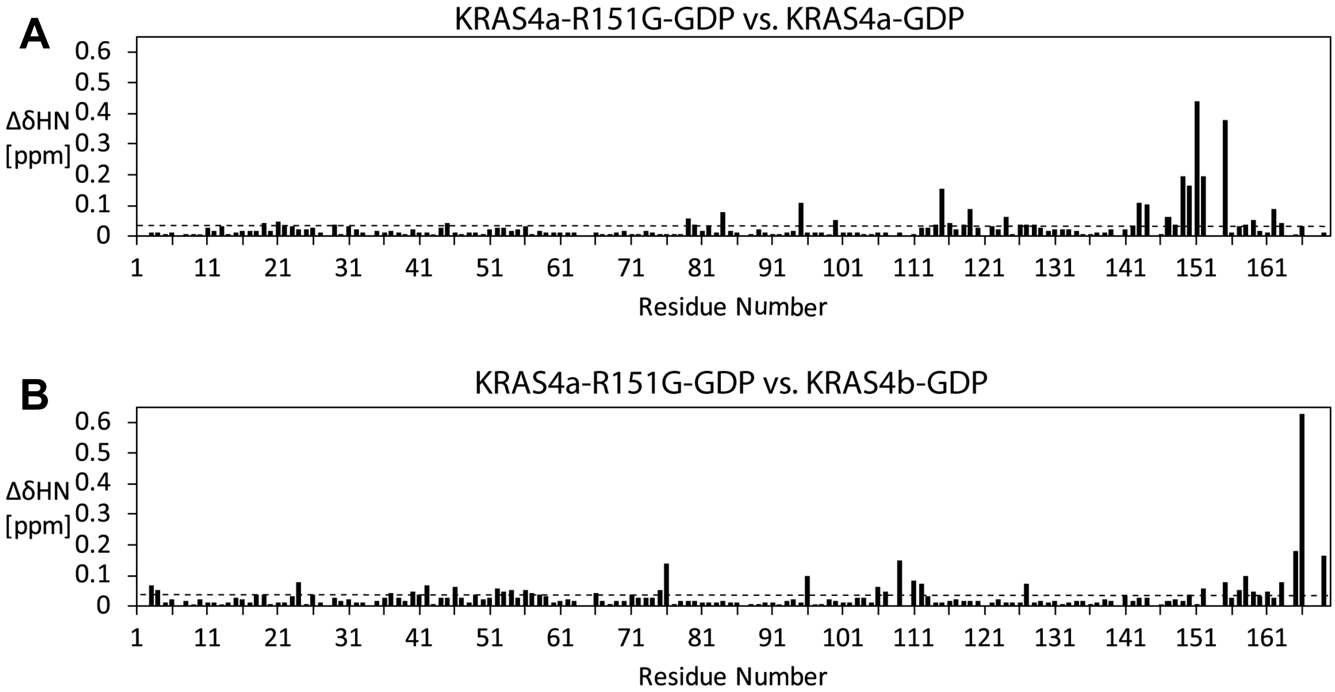

Fig. S3. Chemical shift differences between KRAS4a-R151G and the two isoforms of WT KRAS (residues 1-169). Chemical shift differences of KRAS4a-R151G-GDP versus **A)** KRAS4a-GDP and **B)** KRAS4b-GDP. The dotted cutoff line at 0.04 ppm accounts for minute variations in sample conditions; measurement precision is better than 4 ppb.

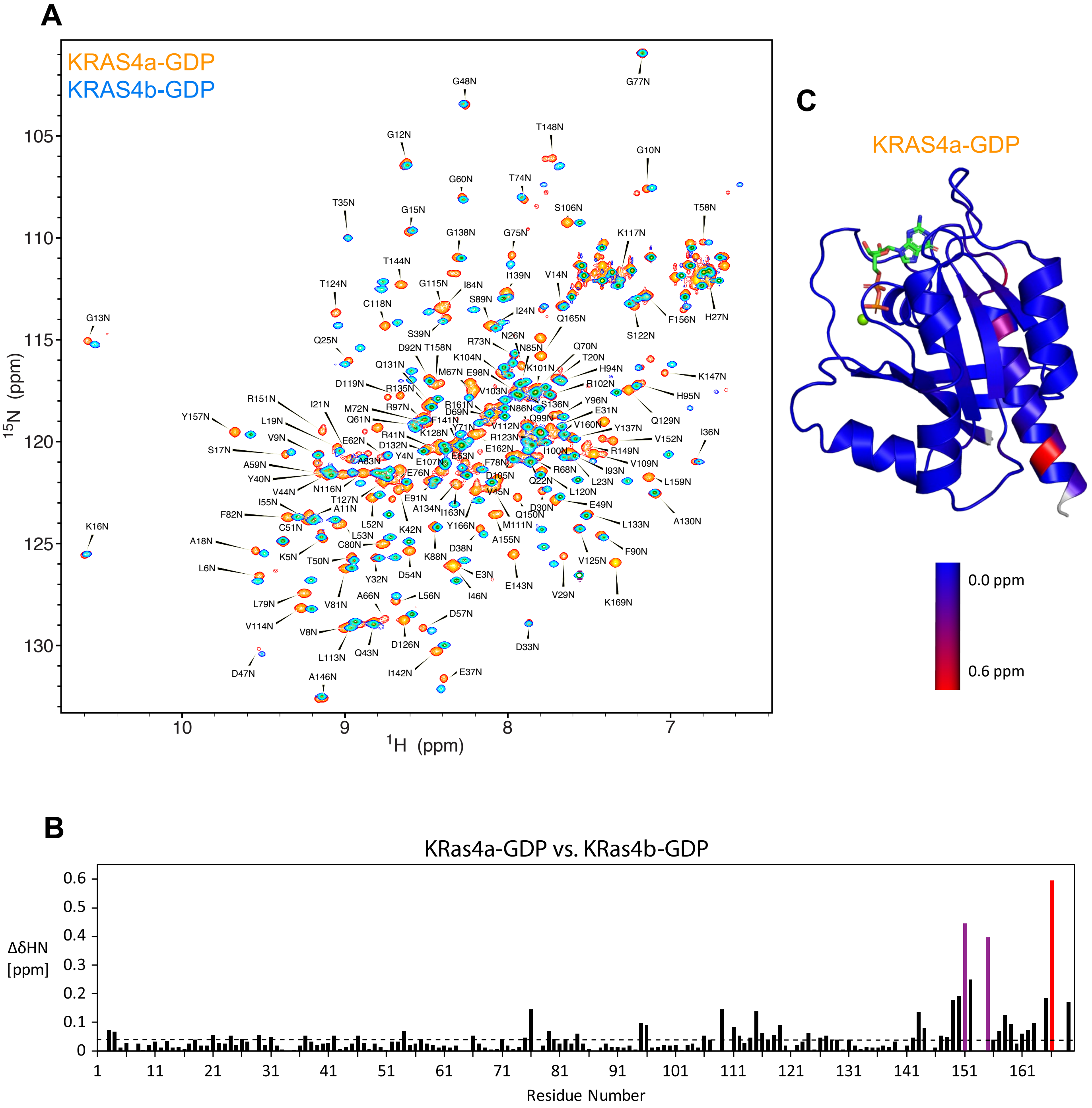

Fig. S4. Amide chemical shift analysis of GDP-bound KRAS isoforms. A) Overlay of ^{1}H-^{15}N HSQC NMR spectra of GDP-bound KRAS4a (orange contours and black sequence assignments) and KRAS4b (blue contours). **B, C)** The KRAS4a vs. KRAS4b amide chemical shift differences plotted along the sequence (B) and their localization on the corresponding crystal structure (C). For KRAS4a-GDP split peaks, only the major peak was used in calculating the amide chemical shift perturbation. The dotted cutoff line at 0.04 ppm accounts for minute variations in sample conditions; measurement precision is better than 4 ppb. The largest CSPs are highlighted with similar colors in B and C panels.

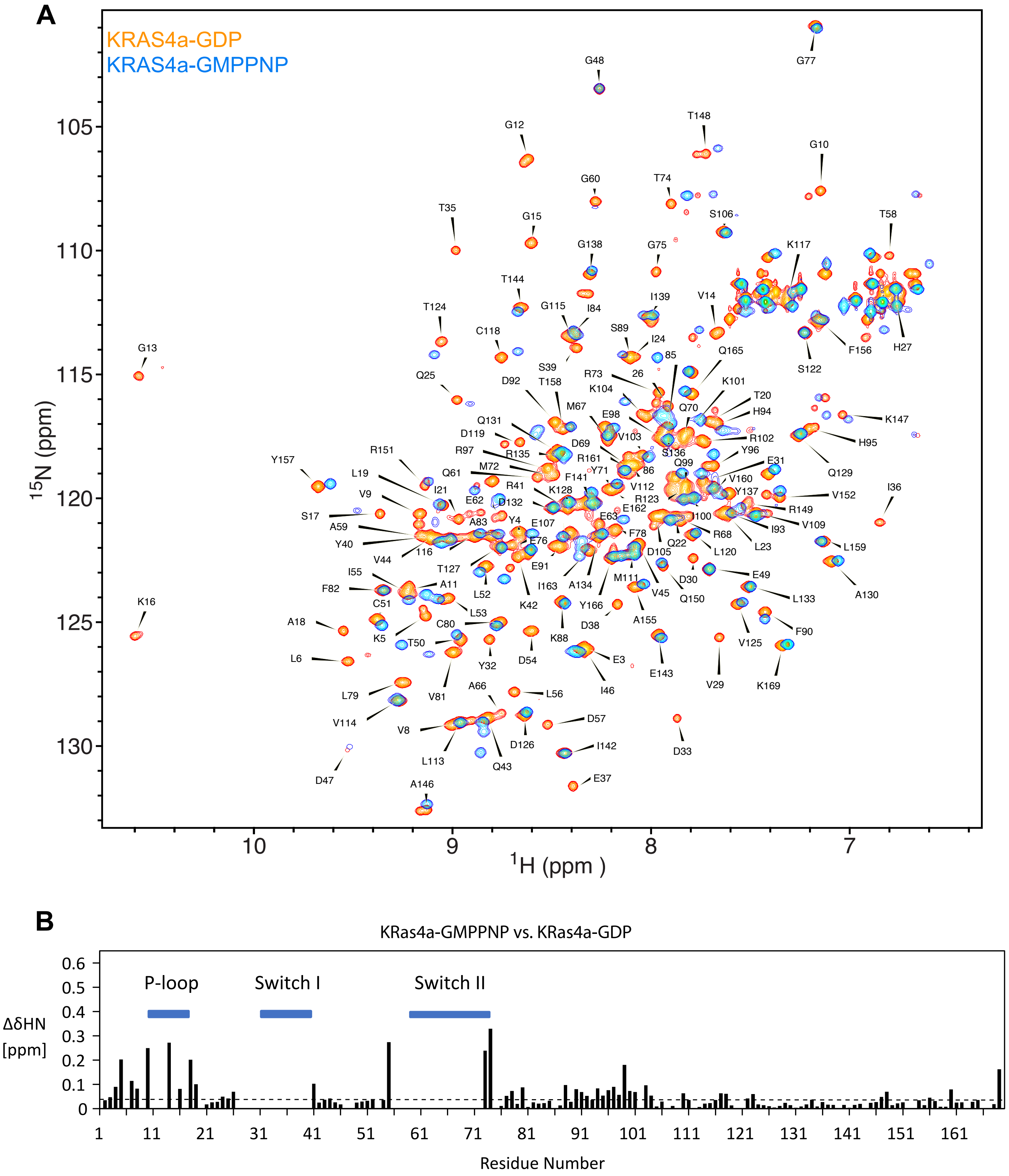

Fig. S5. Amide chemical shift analysis of active and inactive KRAS4a. A) Overlay of the ^{1}H-^{15}N HSQC NMR spectra of KRAS4a-GDP (red contours; amide assignments in black) and KRAS4a-GMPPNP (blue contours). **B)** Amide chemical shift differences plotted along the sequence. For split KRAS4a-GDP resonances, only the major peak was used. The dotted cutoff line at 0.04 ppm accounts for minute variations in sample conditions; measurement precision is better than 4 ppb.

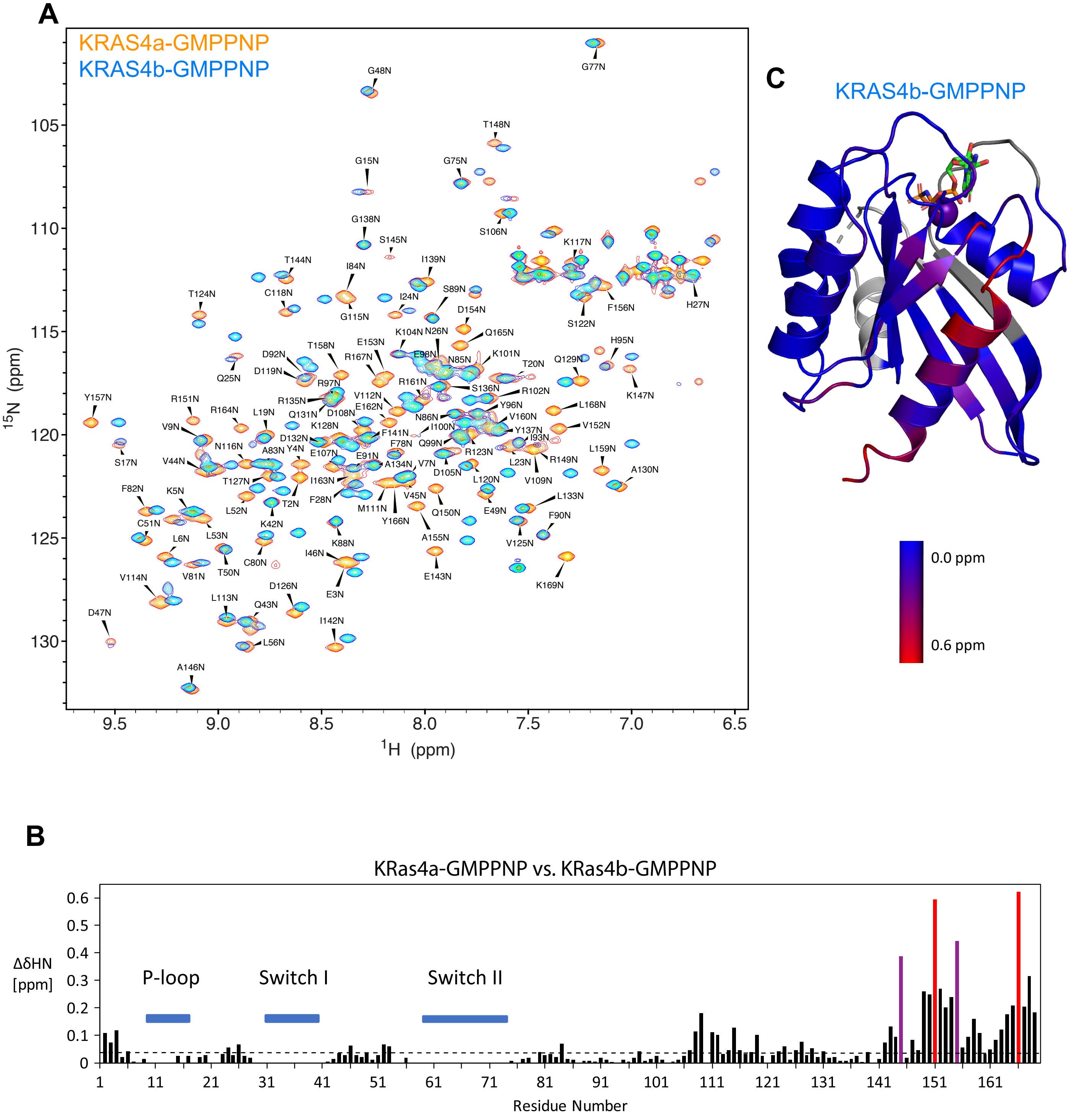

Fig. S6. Amide chemical shift analysis of GMPPNP-bound KRAS isoforms. A) Overlay of 1H-15N HSQC NMR spectra of GMPPNP-bound KRAS4a (orange contours and black sequence assignments) and KRAS4b (blue contours). **B, C)** The KRAS4a vs. KRAS4b amide chemical shift differences plotted along the sequence (B) and their localization on the KRAS4b-GMPPNP crystal structure (C). The exchange broadened dynamic Switch I and II regions in the GMPPNP-loaded form are shown in gray. The dotted cutoff line at 0.04 ppm accounts for minute variations in sample conditions; measurement precision is better than 4 ppb. The largest CSPs are highlighted with similar colors in B and C panels.

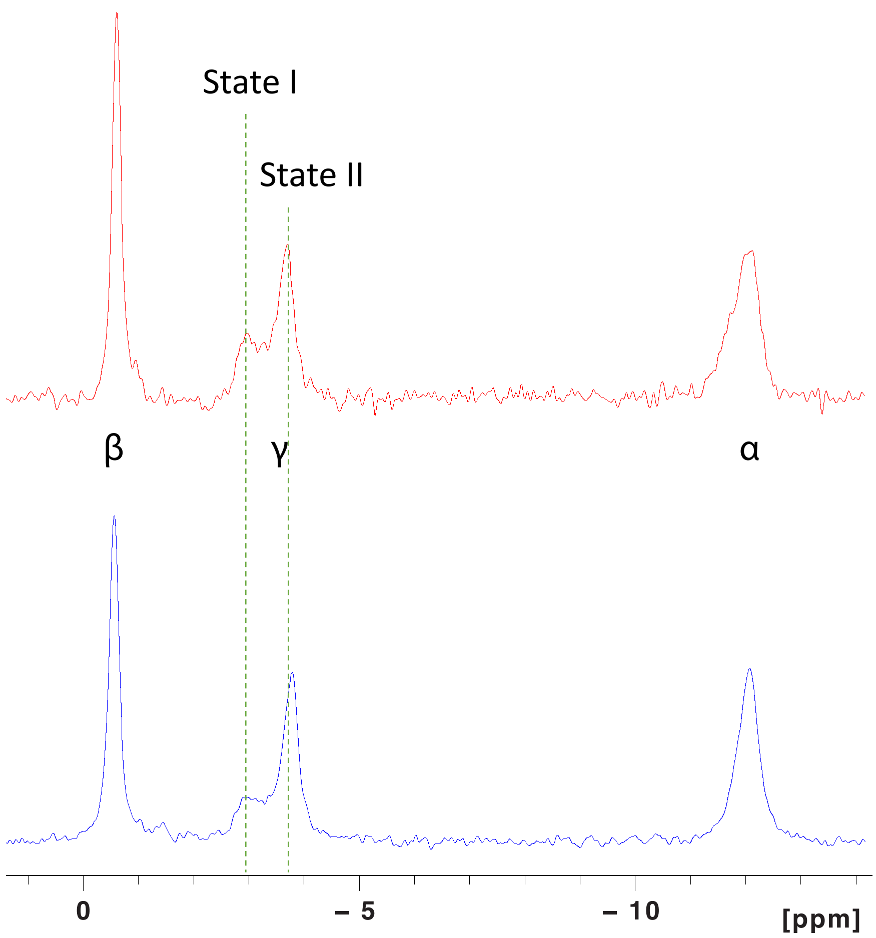

Fig. S7. Aligned 1D ^{31}P spectra of KRAS4a (red) and KRAS4b (blue) in the GMPPNP-bound state at 5 °C.

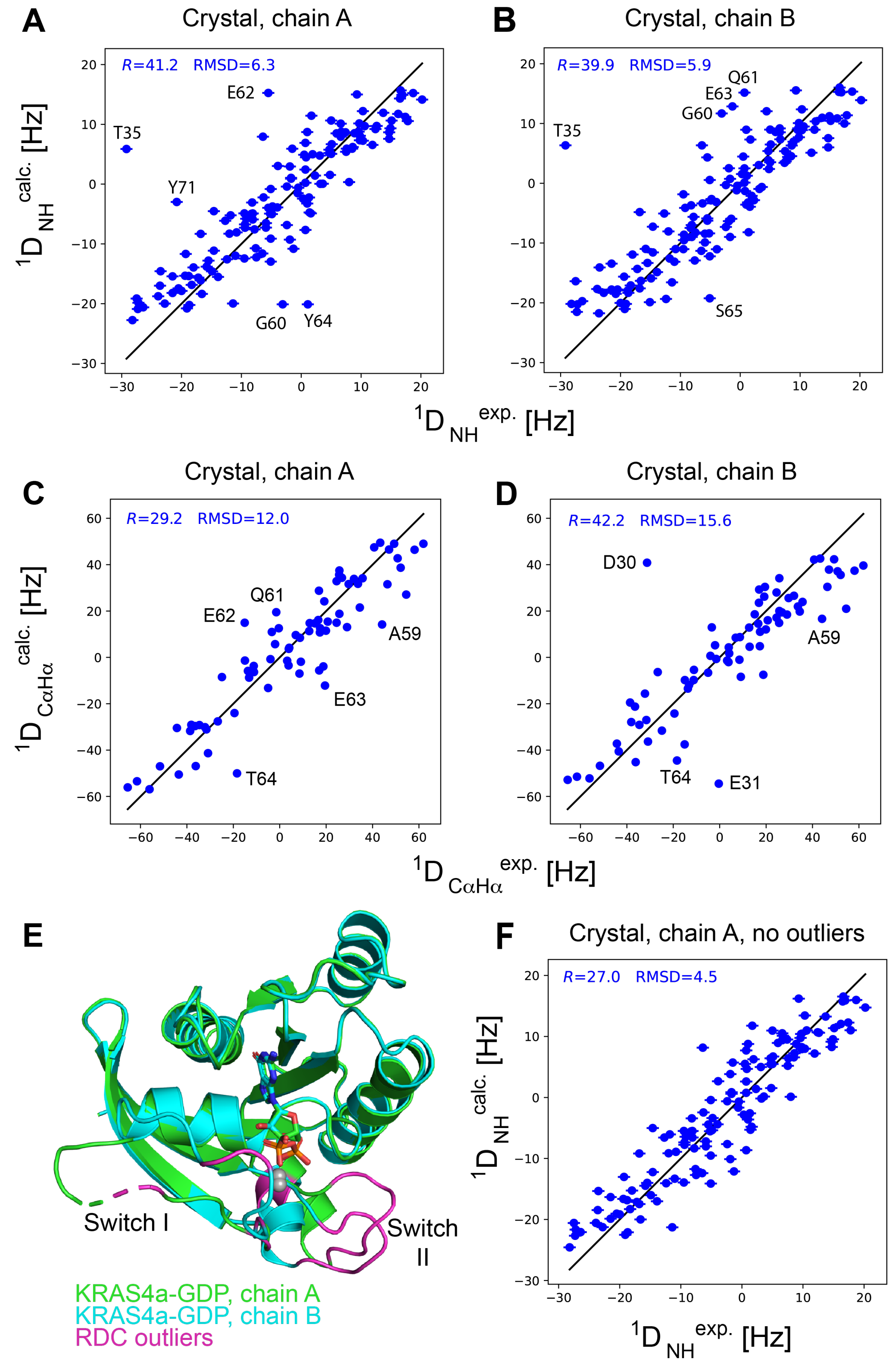

Fig. S8. **RDCs report on crystal packing of flexible loops. A-D)** KRAS4a-GDP Pf1 RDCs fitted to chain A of the KRAS4a-GDP crystal structure (A, C) and to chain B (B, D). **E)** Overlay of the two crystal structures in the asymmetric unit of KRAS4a-GDP (chain A, green and chain B, cyan) with the RDC outliers in magenta (T35, Switches I and II). **F)** KRAS4a-GDP Pf1 RDCs fitted to chain A of the crystal structure, with outliers excluded. In panels A-D and F, the R-factor is given as a percent, and the RMSD is given in Hertz.

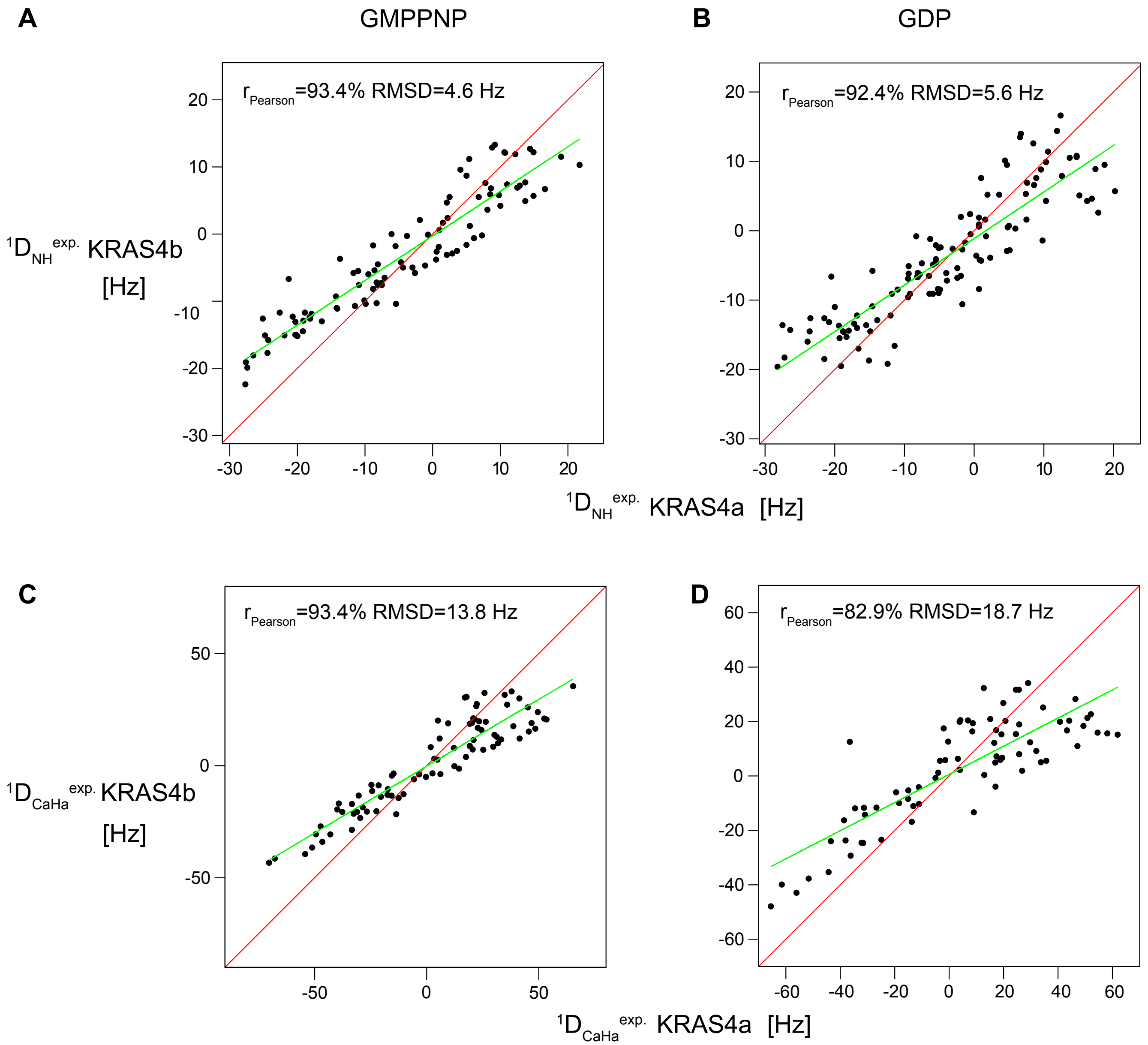

Fig. S9. **Comparison of the Pf1 experimental RDCs of KRAS4a and KRAS4b** Measured $^{1}D_{NH}^{4a}$ vs. $^{1}D_{NH}^{4b}$ in the GMPPNP-bound state **A)** and GDP-bound state. **B)** Measured $^{1}D_{C\alpha H\alpha}^{4a}$ vs. $^{1}D_{C\alpha H\alpha}^{4b}$ in the GMPPNP-bound state **C)** and GDP-bound state **D)**. The slope of the regression lines (green) is different from one (red lines) because of differences in the degree of alignment caused by the slightly unequal concentration of the Pf1 alignment medium in the samples of the two isoforms. The $r_{Pearson}$ are Pearson correlation coefficients.

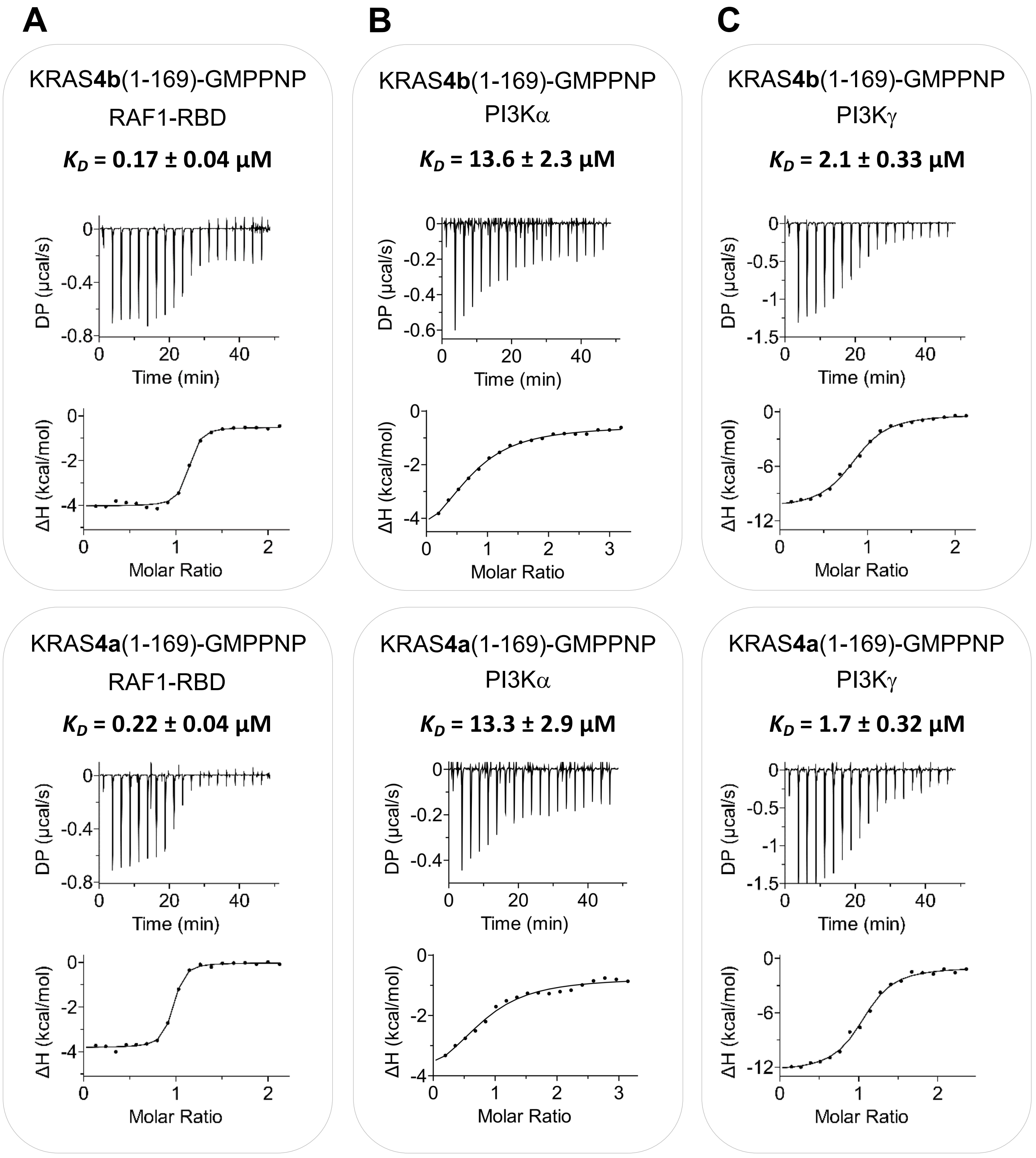

Fig. S10. The G-domain of both KRAS isoforms binds to downstream effectors with similar affinity. Isothermal titration calorimetry experiments to measure the dissociation constant for GMPPNP-bound WT KRAS4a (residues 1-169) or KRAS4b (residues 1-169) with (A) RAF1 RBD, (B) PI3Kα, or (C) PI3Kγ. Differential power (*DP*) is a measure of the energy required to maintain isothermal conditions between the reference cell and the sample cell.

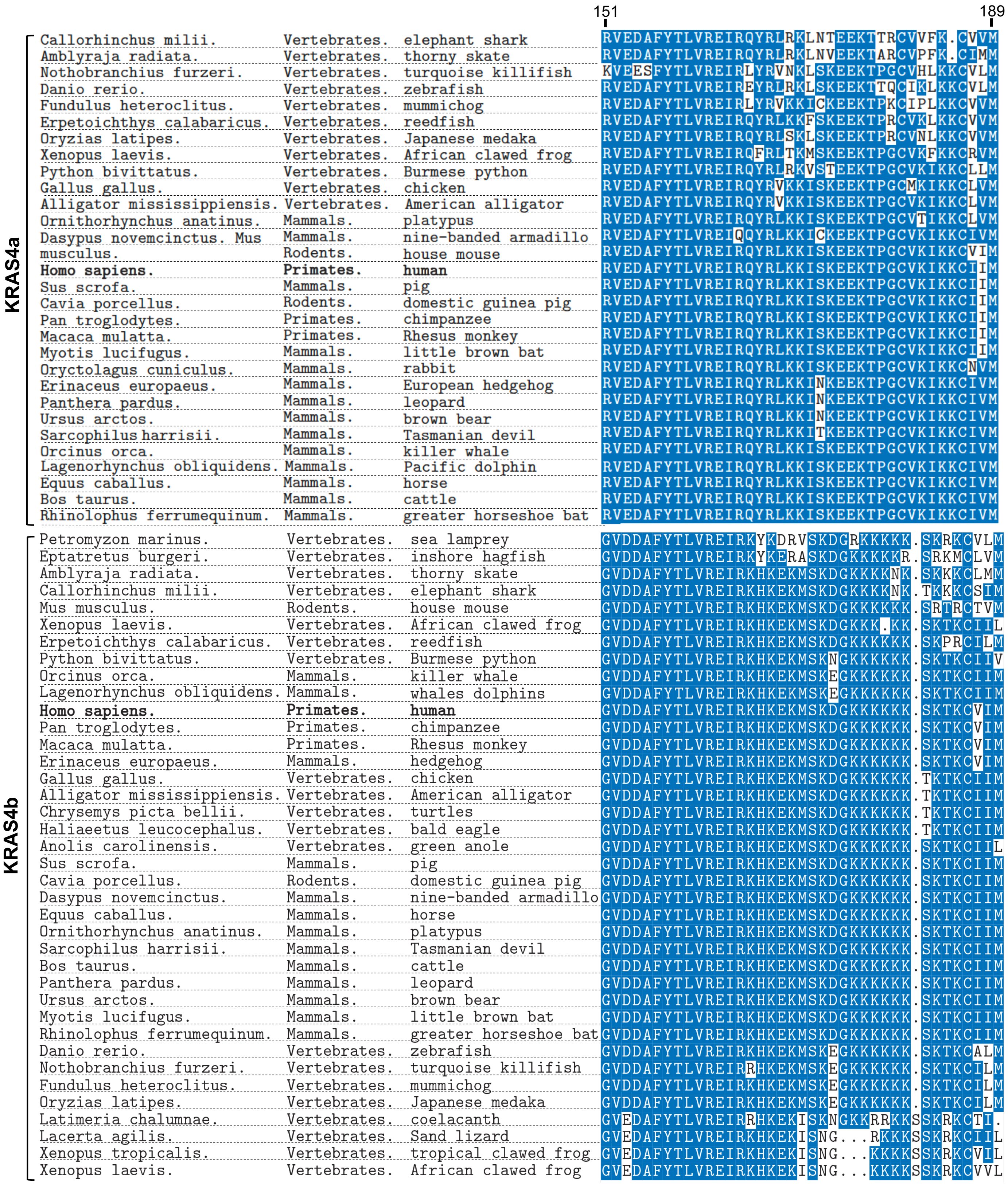

Fig. S11. Alignment of amino acid sequences of exon 4 for two KRAS isoforms from various selected model organisms showing evolutionary conservation. After an initial assessment of full protein sequences of all collected KRAS or KRAS-like sequences in selected model organisms, amino acids corresponding to exon 4 (from residues 151 to 188/189) for various KRAS4a- and KRAS4b-like isoforms were clustered separately and aligned with Clustal Omega using the default settings.

A

	RAF1	
	Bmax	B50
KRAS4a R151G	128.4 ± 4.2	1.1 ± 0.1
KRAS4a WT	139.1 ± 2.7	1.0 ± 0.1

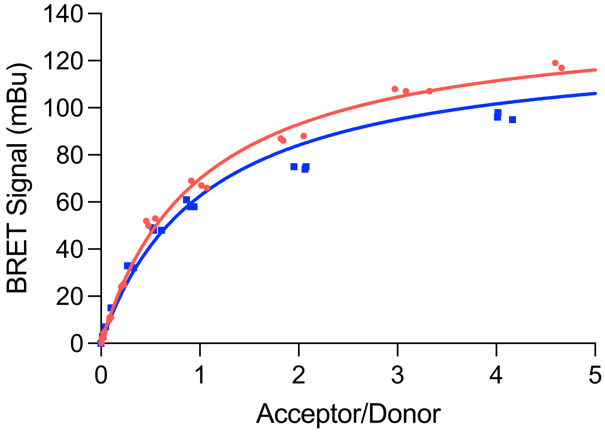

B

	RAF1	
	Bmax	B50
KRAS4b D153E	81.4 ± 2.4	1.3 ± 0.1
KRAS4b WT	57.0 ± 1.8	1.2 ± 0.1

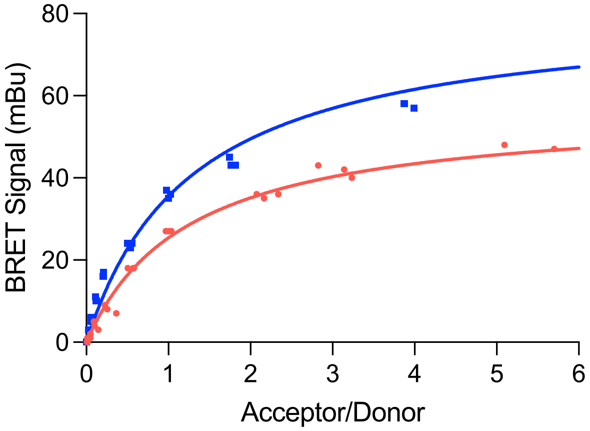

C

	RAF1	
	Bmax	B50
KRAS4a E153D	205.7 ± 9.2	1.7 ± 0.2
KRAS4a WT	148.3 ± 4.1	1.1 ± 0.1

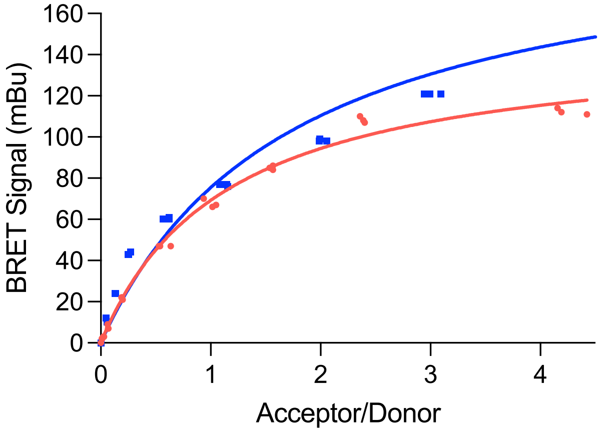

Fig. S12. BRET saturation curve experiments for mVenus-tagged KRAS and NanoLuc-tagged RAF1 to examine the effects of substitution at positions 153 and 151 in the G-domain. BRET saturation curve experiments between mVenus-tagged **(A)** KRAS4a-R151G **(B)** KRAS4b-D153E and **(C)** KRAS4a-E153D mutant with NanoLuc(nL)-tagged RAF1 construct transiently expressed in HEK293T cells. NanoLuc-effector donor construct expression levels were held constant while mVenus-RAS acceptor constructs were titrated, and adjusted BRET signal is reported as a function of acceptor/donor concentration. Data are representative of the four biological replicates. $BRET_{50}$ (B50) and $BRET_{max}$ (Bmax) values from the curve fitting are shown in each panel as the fitted value ± the standard error.

Supplementary Tables

Table S1: Fitted Bmax and B50 values for the individual biological replicates of the BRET experiments reported in Figure 8 and Figure S12.

	**PI3Kα**							
	**Biol. Replicate 1**		**Biol. Replicate 2**		**Biol. Replicate 3**		**Biol. Replicate 4**	
	**Bmax**	**B50**	**Bmax**	**B50**	**Bmax**	**B50**	**Bmax**	**B50**
**KRAS4a**	49.4	2.69	48.6	1.29	49.4	2.69		
**KRAS4b**	26.7	1.27	25.4	1.36	26.7	1.27		
**4a (G-domain) + 4b (HVR)**	32.6	0.91	26.0	0.67	26.4	0.92		
**4b (G-domain) + 4a (HVR)**	56.7	1.25	47.0	1.48	43.1	1.16		
	**RAF1**							
	**Biol. Replicate 1**		**Biol. Replicate 2**		**Biol. Replicate 3**		**Biol. Replicate 4**	
	**Bmax**	**B50**	**Bmax**	**B50**	**Bmax**	**B50**		
**KRAS4a**	88.9	1.13	80.6	5.43	79.4	1.18		
**KRAS4b**	53.2	1.15	22.2	0.50	51.3	1.05		
**4a (G-domain) + 4b (HVR)**	57.9	1.21	77.8	0.84	72.0	1.04		
**4b (G-domain) + 4a (HVR)**	61.7	0.88	82.0	0.67	83.2	0.67		
**KRAS4b D153E**	74	1.24	82	1.49	81	1.29	116	2.05
**KRAS4b**	51	0.83	66	1.4	57	1.24	88	1.01
**KRAS4a E153D**	181.0	2.62	183.0	1.69	206.0	1.73	254.0	4.67
**KRAS4a**	129.0	0.94	147.0	1.27	148.0	1.14	144.0	1.45
**KRAS4a R151G**	128	1.05	136	1.34	164	2.05	171	2.74
**KRAS4a**	139	0.99	150	1.12	160	1.38	151	1.28

Table S2: p-values resulting from paired t-tests for significant differences in BRET Bmax or B50 values for the individual biological replicates depicted in Figure 8 and Figure S12. A p-value < 0.05 is considered significant.

	RAF1		PI3Kα	
	**p-value (Bmax)**	**p-value (B50)**	**p-value (Bmax)**	**p-value (B50)**
**KRAS4a vs KRAS4b**	0.0463	0.4099	<0.0001	0.2054
**4a (G-domain) + 4b (HVR) vs 4b (G-domain) + 4a (HVR)**	0.4818	0.0527	0.0012	0.0449
**KRAS4b vs KRAS4b D153E**	0.0028	0.1811		
**KRAS4a vs KRAS4a E153D**	0.0281	0.1058		
**KRAS4a vs KRAS4a R151G**	0.9765	0.1510		

Table S3: Details of constructs used for BRET assay.

Clone ID	Clone Name	Entry clone 1	Entry clone 2	Entry clone 3	Vector
R713-M62-305	NanoLuc-PIK3CA	CMV51 (Addgene 162973)	NanoLuc (Addgene 162938)	Hs.PIK3CA (Addgene 70447)	pDest-305
R718-M15-313	RAF1-NanoLuc	CMV51 (Addgene 162927)	Hs.RAF1 (Addgene 70498)	NanoLuc (Addgene 162901)	pDest-313
R783-M70-303	mVenus-Hs.KRAS4b(1-168)-Hs.KRAS4a(169-189)	CMV51 (Addgene 162973)	mVenus (Addgene 162943)	ATUM, Inc.	pDest-303
R783-M71-303	mVenus-Hs.KRAS4a(1-168)-Hs.KRAS4b(169-188)	CMV51 (Addgene 162973)	mVenus (Addgene 162943)	ATUM, Inc.	pDest-303
R714-M32-304	mVenus-Hs.KRAS4a	CMV51 (Addgene 162973)	mVenus (Addgene 162943)	Hs.KRAS4a (Addgene 83166)	pDest-304
R718-M86-305	mVenus-KRAS4b	CMV51 (Addgene 162973)	mVenus (Addgene 162943)	Hs.KRAS4b (Addgene 83129)	pDest-305
R750-M85-303	mVenus-tev-Hs.KRAS4b	CMV51 (Addgene 162973)	mVenus (Addgene 162943)	PCR	pDest-303
R750-M86-303	mVenus-tev-Hs.KRAS4a	CMV51 (Addgene 162973)	mVenus (Addgene 162943)	PCR	pDest-303
R919-M01-303	mVenus-tev-Hs.KRAS4a(1-189) R151G	CMV51 (Addgene 162973)	mVenus (Addgene 162943)	Mutagenesis	pDest-303
R919-M02-303	mVenus-tev-Hs.KRAS4a(1-189) E153D	CMV51 (Addgene 162973)	mVenus (Addgene 162943)	ATUM, Inc.	pDest-303
R919-M04-303	mVenus-tev-Hs.KRAS4b(1-188) D153E	CMV51 (Addgene 162973)	mVenus (Addgene 162943)	ATUM, Inc.	pDest-303
